# Release systems based on self-assembling RADA16-I hydrogels with a signal sequence which improves wound healing processes

**DOI:** 10.1038/s41598-023-33464-w

**Published:** 2023-04-18

**Authors:** Maria Dzierżyńska, Justyna Sawicka, Milena Deptuła, Paweł Sosnowski, Piotr Sass, Barbara Peplińska, Zuzanna Pietralik-Molińska, Martyna Fularczyk, Franciszek Kasprzykowski, Jacek Zieliński, Maciej Kozak, Paweł Sachadyn, Michał Pikuła, Sylwia Rodziewicz-Motowidło

**Affiliations:** 1 Department of Biomedical Chemistry, Faculty of Chemistry, University of Gdańsk, Gdańsk, Poland; 2 Laboratory of Tissue Engineering and Regenerative Medicine, Department of Embryology, Medical University of Gdańsk, Gdańsk, Poland; 3 Laboratory for Regenerative Biotechnology, Faculty of Chemistry, Gdańsk University of Technology, Gdańsk, Poland; 4 NanoBioMedical Centre, Adam Mickiewicz University, Poznań, Poland; 5 Department of Macromolecular Physics, Faculty of Physics, Adam Mickiewicz University, Poznań, Poland; 6 Department of Surgical Oncology, Medical University of Gdańsk, Gdańsk, Poland

**Keywords:** Medicinal chemistry, Drug delivery, Peptides, Biomaterials, Biomedical materials, Drug delivery

## Abstract

Self-assembling peptides can be used for the regeneration of severely damaged skin. They can act as scaffolds for skin cells and as a reservoir of active compounds, to accelerate scarless wound healing. To overcome repeated administration of peptides which accelerate healing, we report development of three new peptide biomaterials based on the RADA16-I hydrogel functionalized with a sequence (AAPV) cleaved by human neutrophil elastase and short biologically active peptide motifs, namely GHK, KGHK and RDKVYR. The peptide hybrids were investigated for their structural aspects using circular dichroism, thioflavin T assay, transmission electron microscopy, and atomic force microscopy, as well as their rheological properties and stability in different fluids such as water or plasma, and their susceptibility to digestion by enzymes present in the wound environment. In addition, the morphology of the RADA-peptide hydrogels was examined with a unique technique called scanning electron cryomicroscopy. These experiments enabled us to verify if the designed peptides increased the bioactivity of the gel without disturbing its gelling processes. We demonstrate that the physicochemical properties of the designed hybrids were similar to those of the original RADA16-I. The materials behaved as expected, leaving the active motif free when treated with elastase. XTT and LDH tests on fibroblasts and keratinocytes were performed to assess the cytotoxicity of the RADA16-I hybrids, while the viability of cells treated with RADA16-I hybrids was evaluated in a model of human dermal fibroblasts. The hybrid peptides revealed no cytotoxicity; the cells grew and proliferated better than after treatment with RADA16-I alone. Improved wound healing following topical delivery of RADA-GHK and RADA-KGHK was demonstrated using a model of dorsal skin injury in mice and histological analyses. The presented results indicate further research is warranted into the engineered peptides as scaffolds for wound healing and tissue engineering.

## Introduction

According to the 2021 statistics of the World Health Organization, more than 20 million people around the world are affected by the problem of hard-to-heal skin tissue wounds. Wound healing is a dynamic process consisting of overlapping phases, including hemostasis, inflammation, proliferation and remodelling^1^. During wound healing a number of types of cells are activated, namely keratinocytes, dermal fibroblasts and cells of the immune system, including neutrophils, lymphocytes, and mast^2^. From a biological and clinical point of view, the critical stage of wound healing is the closure of the epithelial gap, primarily associated with forming an epidermis (epithelialization) on the entire surface of the wound, and the reconstruction of the wound dermis^3^. Skin repair is regulated and stimulated by growth factors, cytokines and the extracellular matrix, but the process, despite its complexity, cannot restore skin to the state it was in prior to injury^4^. Currently, many methods of wound treatment are available, but their effects are not very effective in the treatment of chronic wounds, which include, for example, diabetic foot ulcers^5^. Among the many dressings in recent times, hydrogels are the most widely used. They have many advantages as wound dressings. Primarily, they facilitate a humid microenvironment while simultaneously absorbing wound exudate. Maintaining local environment is wiedly regarded as a crucial factor for achieving effective wound healing, regardless of the underlying cause of the wound^5^. Hydrogels offer protection to the wound against the external environment, akin to the conventional dressings. In addition, they exhibit adhesion and hemostatic properties, while still allowing for airflow within the wound site^6^. Due to its structure, they are an excellent scaffold for proliferating and migrating skin cells during wound healing. Hydrogels also have the ability to encapsulate bioactive agents and drugs in its 3D scaffold to aid during the healing process, which makes them excellent drug delivery systems^7–10^.

Hydrogels as scaffolds for cells should be biocompatible, cause no cytotoxic effects, and induce no immunological responses such as inflammation or allergic reactions^11^. Their structure should allow the penetration of nutrients and growth factors available for absorption by cells contained in the scaffolds^12^. Viable released cells can then rebuild the epidermis and dermis through proliferation and by releasing growth factors that paracrinally stimulate wound healing.

One of the classes of compounds that form biocompatible hydrogel scaffolds is self-assembling peptides (SAPs). They are part of a modern biomedical approach to advanced wound healing that aims to isolate the wound from the environment, activate regeneration and provide scaffolds to support skin reconstruction. Following this serendipitous discovery^13^, SAPs became recognizable as biomaterials. They undergo spontaneous arrangement, leading to the formation of stable secondary structures (β-sheet, α-helix) and then further to fibres, fibrils or gels^14^. The properties of SAPs make them ideal scaffolds. SAPs present several advantages over conventional scaffolds in tissue engineering applications. Firstly, they exhibit spontaneous formation of well-defined nanostructures and hierarchical supramolecular assemblies that closely resemble the extracellular matrix (ECM) of natural tissues^15^. This provides a biologically relevant environment that is favorable for cellular attachment, proliferation, and differentiation^16,17^. Secondly, SAPs can be designed with specific bioactive sequences that can interact with cell receptors, promoting various cellular activities, including adhesion, migration, and differentiation^17^. Moreover, SAPs can be engineered to possess specific mechanical properties such as stiffness and elasticity, which are essential for directing cellular behavior and tissue development^18^. Thirdly, SAPs are highly biocompatible and biodegradable, making them ideal for use in regenerative medicine^19,20^. They can be readily modified with functional groups to promote specific biological activities, and their degradation products are non-toxic and can be metabolized by the body without adverse effects^21^.

A self-assembling group of oligopeptides, which belong to a generation of hydrogels which form ionic self-complementary β-sheets, are the RADA16 peptides^22^. These consist of periodic repeats of the RADA sequence, where positively charged arginine and negatively charged aspartic acid alternate with each other, resulting in the formation of the peptide Ac-RADARADARADARADA-NH_2_ (RADA16-I). Alanine, as a hydrophobic amino acid with hydrophilic residues, is believed to induce the formation of the beta-sheet^23^. Gelation occurs spontaneously a couple of hours after dissolving this peptide in water or can be accelerated by adjusting the pH to neutral or by adding a buffer solution. Interestingly, RADA16-I has the capacity to reassemble to the same structure after sonication. Sonication presumably breaks some of the weak bonds while not affecting the β-sheet structure^24^.

The peptide RADA16-I itself has intrinsic properties which are beneficial for differential cell growth, proliferation and migration, and which influence wound healing processes^25–28^. Moreover recently RADA16-I peptide was studied as a artificial ECM for studying cancer cell behaviours^29^. Delivery of an unmodified RADA16-I hydrogel results in immediate hemostasis, which has been proven by its application on cut wounds on the brain, liver, femoral artery, spinal cord, skin and ischemia–reperfusion injury^30–33^. The results showed that RADA16-I alone slightly increased wound closure and reepithelization compared to the control. Enhanced wound healing has also been observed in surgical periodontal defects after application of RADA16-I hydrogel^34^. The authors indicate an increase in cell recruitment and angiogenesis in rats four weeks after surgery.

As RADA16-I is a peptide there is a possibility of covalently elongating it with different bioactive peptides without, to any great extent, disturbing its gelation properties. Successful modifications that have incorporated different peptide epitopes to the RADA16-I sequence while maintaining its gel properties are not only the short four-aa chain peptide RGDS, designed for periodontal therapy^35^, but also longer 12-aa^36^ or 14-aa chain peptides such as TAGSCLRKFSTMGG, which has been applied for enhancement of endothelial cell adhesion^37^. Addition of a functional bioactive peptide can induce beneficial properties for wound healing.

In this work, we report a strategy to prepare peptide hydrogels that enhance wound healing processes. We have conjugated short bioactive peptide motifs (GHK^38^, KGHK^39^ and RDKVYR^40^) to a RADA16-I parent molecule through an elastase-cleaved sequence, AAPV^41^ and two triple-glycine linkers (GGG). GHK, KGHK and RDKVYR have been proven to be factors which accelerate wound healing processes^38–40,42^. GHK and KGHK are capable of modulating the activity of metalloproteinases as well as their inhibitors, TIMP-1 and TIMP-2, which play crucial role in the regulation of tissue remodeling and wound healing processes. Therefore, KGHK and GHK function as a primary regulatory factors by controlling the balance between the activity of metalloproteinase and their inhibitors, which is necessary for proper tissue repair and maintenance of skin integrity. Further investigations have revealed that GHK exhibits properties that enable it to selectively recruit both immune and endothelial cells to the tissue injury^38^. KGHK peptide is capable of eliciting a stimulatory effect on the formation of an intricate network of endothelial cells, a critical process for the establishment and maintenance of proper tissue vasculature^39^. Both peptides promote proliferation of fibroblasts^39,43^. RDKVYR peptide, also known as Imunofan (IM) was recently proven to stimulate migration of keratinocytes and treated wounds seems to be richer in fibroblasts in comparison to control. Also adipose-derived stem cell cultures seemed to have increases in expression of *POU5F1* gene, which encodes *OCT4* protein, which is responsible factor for unraveling the regulatory circuit responsible for inducting and maintaining cell pluripotency^40,44^. The designed peptides contain a bioactive and a self-assembling motif and form a stable hydrogel in aqueous solution.

The peptide hybrids presented in this work are enzyme-triggered controlled release systems, the structural, chemical and physicochemical properties of which were examined using multiple methodological approaches, including circular dichroism, thioflavin T assay, transmission electron microscopy, atomic force microscopy, scanning electron cryomicroscopy and rheology. The peptides were tested for cytotoxic and pro-proliferative properties on cultured keratinocytes and fibroblasts and, finally, they were applied topically to examine the effects on skin wound healing in mice.

## Materials and methods

### Peptide synthesis, purification and counterion exchange

**Figure 1:**
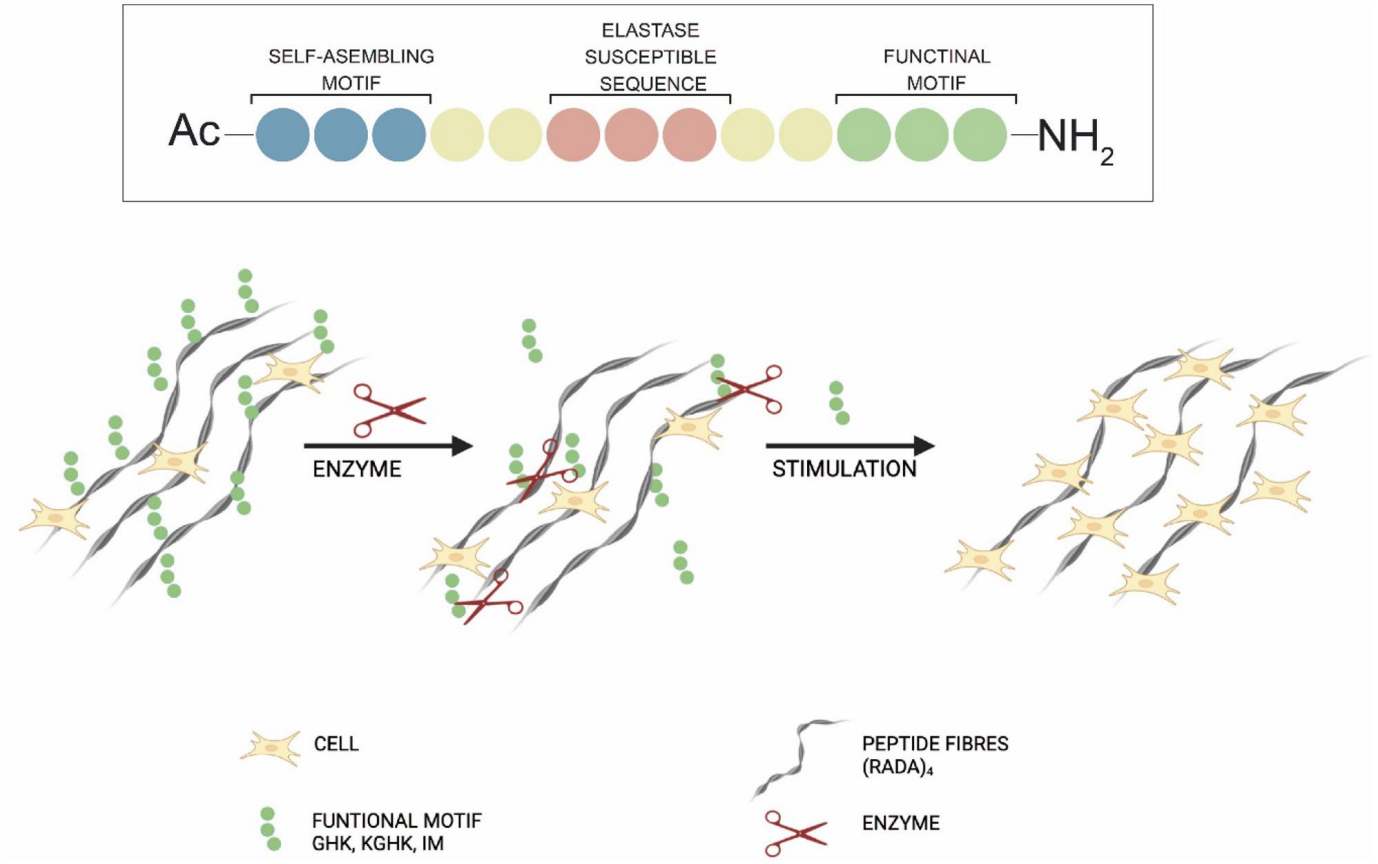
Schematic illustration model of the designed peptides.

All peptide syntheses were performed using standard Fmoc-based solid-phase peptide synthesis (SPPS) protocols on an automated Microwave Peptide Synthesizer (Liberty Blue, CEM Corporation). TentaGel R RAM was used as a solid resin, and DIPEA, DIC and Oxyma pure were used as coupling reagents. 20% piperidine in DMF was used to cleave Fmoc, and *N*-acetylimidazole was used as a capping reagent. Deprotection was performed in a mixture of Trifluoroacetic acid/Phenol/Triisopropylsilane/H_2_O (88:5:5:2 v/v/v/v). Peptides were purified by HPLC using a semi-preparative Jupiter Proteo (Phenomenex) column C12 4 µm, 90 Å with solvents containing TFA as an additive. The trifluoroacetate counterion of all peptides was exchanged for the acetate on SPE columns. The peptides were characterized by HPLC and LCMS (Supplementary Data—Table 1S, 2S and Fig. 1S).

### Gel formations of peptide hybrids

The peptide gels were obtained by dissolving in water to concentration of 10 mg/ml (1%). Before incubation in thermoshaker for a least 4 h, the peptides were vortexed well (or sonicated if needed). The pH solution of hybrids were adjusted to 5.5. Between pH 4–8 no precipitation was being observed. The pH was controlled by non-bleeding pH strips. For experiments of elastase enzymatic clevage, circular dichroism measurement, thioflavin T test, stability in water and plasma, affinity, XTT and LDH assay the peptides gels were formed and then diluted to the proper concentration. In AFM and TEM experiments gels (1%) were formed by addition of PBS and then diluted to a proper concentration. For rheological measurements, cryo-SEM and mice experiments gels in concentration of 10 mg/ml was applied with no further dilution. In case of experiments with stability in water and plasma, with cell lines and for mice experiments gels were prepared under sterile conditions.

### Elastase enzymatic cleavage

Human neutrophil elastase (EC No. 3.4.21.37, Sigma-Aldrich) was used to study peptide susceptibility to that enzyme. Peptides were dissolved in 0.1 M Tris HCl, 500 mM NaCl, 0.05% Triton X-100, 20 mM CaCl_2_ pH 7.5 buffer mixture. Peptides (final concentration 1 mM) were mixed with the enzyme (final concentration 150 nM) and incubated for 30 min. in a thermoshaker (37 °C, 300 rpm). The reaction was stopped by a 10% aqueous solution of TFA. The results were analyzed using HPLC and MALDI or LC–MS. Details of this experiment are described in the Supplementary Data.

### Circular dichroism (CD) measurement

The molar ellipticity of peptides were registered on a Jasco J-815 spectrometer (JASCO, Easton, MD, USA). Solutions of peptide hybrids were prepared in water solution (10 mg/ml), incubated at 37 °C for 4 h and then diluted to a final concentration of 0.20 mg/ml. CD spectra measurements were performed at 25 °C, 30 °C, 35 °C, 40 °C, 45 °C, 50 °C, 55 °C and 60 °C, at 190–260 nm wavelength and at a scan speed of 2 cm/min. The background of the solvent was subtracted. The results are presented as the relationship of mean residue molar ellipticity (MRME) [deg × cm^2^ × dmol^−1^] to wavelength [nm]. Measurements were performed in triplicate. In order to determine the peptides’ denaturation temperatures, the molar ellipticity rotatory angle at a fixed wavelength of 196 nm, [θ]_196_, was measured as a function of temperature (max 100 °C). CD temperature measurements were carried out up to a temperature of 100 °C. Above this temperature, the cure was extrapolated with GraphPad Prism 8 software using the 4PL curve fit function. The denaturation temperature was determined as the temperature at which the change in ellipticity (θ) was half complete.

### Thioflavin T (ThT) test

1% gel solutions were prepared in water and incubated. A stock of Thioflavin T (ThT) was prepared in water. Peptides and Thioflavin T were mixed in 96-well plates (COSTAR Flat plate Black) with final concentrations of peptide of 1 mg/ml and 10 µM of ThT. The fluorescence spectra were measured from 455 to 600 nm at a scan rate of 100 nm/min. The excitation wavelength was 420 nm. All fluorescent spectra were recorded using an Infinite 200 Pro (Tecan) plate reader. Experiments were performed in triplicate.

### Rheology

Rheological parameters of the hydrogels were identified using an MCR 302 rheometer (Anton Paar GmbH, Graz, Austria). The rheometer was equipped with a CP50-1 stainless steel cone-and-plate system of 50 mm diameter, 1° cone angle and 50 µm cone truncation and a Peltier controlled temperature unit. An aliquot of 900 µL of 1% hydrogel was applied to a plate cone. The linear viscoelastic (LVE) region was determined through an amplitude sweeps: shear strain (0.01–100% at 6 rad s^−1^) and shear stress (0.001–10 Pa at 6.28 rad s^−1^). Frequency sweep were measured from 0.01 to 100 rad s^−1^ at 2.5% strain. The frequency dependence of the storage modulus (G’) and loss modulus (G”) of the hydrogels were measured at 37 °C. All experiments were carried out in triplicate and reported as the mean of three repeats.

### Atomic force microscopy (AFM)

1% gel solutions were prepared and incubated in PBS. The samples were then diluted to 0.001% concentration. The samples (5 μl) were adsorbed on a freshly cleaved mica surface, rinsed after 30 s. with 100 µl MilliQ water and left to dry (1 h, rt) in the air before visualization. The topography of the deposited samples was analyzed using a JPK NanoWizard® 4 atomic force microscope. The measurements were conducted in QI (Quantitative Imaging) mode using Tap150AL AFM cantilevers (Ted Pella, Inc., Redding, USA). The AFM images were processed and analyzed using the JPK Data Processing software (version spm-6.1.42).

### Transmission electron microscopy (TEM)

1% gel solutions were prepared and incubated in PBS. The samples were then diluted to 0.001% concentration. A drop of solution was applied to a microcellulose membrane and stained with a 1.5% solution of uranyl acetate. The images were registered on a Tecnai Spirit BioTwin FEI Transmission Electron Microscope operating at 120 kV.

### Scanning electron cryomicroscopy (cryoSEM)

1% gel solutions were prepared and incubated. Images were taken using a JEOL JSM-7001F TTLS scanning electron microscope (JEOL Ltd., Japan) equipped with a PP3000T cryoSEM preparation system, which allows cryo specimens to be prepared, processed and transferred into the SEM chamber. The sample was rapidly frozen by plunging the holder with droplets of hydrogels (1%) into slush nitrogen (temperature about − 210 °C) and transferred under vacuum to the preparation chamber mounted on the SEM. The specimen was fractured inside the preparation chamber, at − 185 °C, to expose a fresh surface. It was then sublimated and coated with a thin platinum layer. Finally, the sample was loaded under vacuum into the SEM cryo stage (− 190 °C) where the surface was imaged by applying an accelerating voltage of 5 kV and a secondary electron (SEI) detector.

### Stability in water and presence in human plasma

Blood was drawn from healthy volunteers, and lithium/heparin tubes were used to remove the clot by centrifugation. The plasma was aliquoted and incubated with the desired compounds for 24 h at 37 °C, at final concentrations of 5.83 µM for RADA, 3.12 µM for RADA-IM, 3.76 µM for RADA-KGHK and 3.52 µM for RADA-GHK using a ratio of 4 parts of plasma to 1 part peptide. The samples were prepared under sterile conditions following the protocol previously described^45^. The samples were collected at six time points (0, 1, 2, 3, 6 and 24 h) for determining stability in water solutions, and at eleven time points (0, 10, 20, 30, 40, 50 and 60 min., and 2, 3, 6 and 24 h) for determining stability in human plasma. The analyses were performed by HPLC using Phenomenex Luna C18(2) columns (5 µm, 100 Å 4.6 × 250 mm) monitored by a PDA detector. For calculations, peak areas were compared to a calibration curve calculated by Shimadzu LCsolution Software. Additional peak detection was performed by LC–MS mass spectrometry using a Kromasil C8 column (5 µm, 90 Å 1.0 × 250 mm). Details of the peptide stability procedure are described in the Supplementary Data.

### Affinity test

Immobilization of protein on a microcolumn and affinity tests were performed as described previously by *Spodzieja et al.*^46^. Peptides were added onto a prepared albumin-Sepharose column equilibrated in ammonium hydrogen carbonate, pH 7.4, and incubated for 2 h at 25 °C. After 2 h, the column was washed with ammonium hydrogen carbonate, collecting the first and the last milliliter of the solution (supernatant and last wash fraction). The peptide complexes with albumin were dissociated by 15 min. incubation in 0.1% TFA, with gentle shaking. The procedure was repeated twice, and the elution fractions were collected. The collected fractions were lyophilized and analyzed using mass spectrometry (MALDI, Brucker).

### Isolation of primary dermal fibroblasts

Skin samples were obtained from patients of the Oncological Surgery Clinic in the Medical University of Gdańsk. The procedure was approved by the Independent Bioethics Commission for Research of the Medical University of Gdańsk (NKBBN/387/2014) and written informed consent was obtained from patients prior to surgery. All experiments were performed in accordance with guidelines and regulations. The isolation of fibroblasts was performed by explant culture as previously described^40^. Briefly, skin samples were cut into small pieces, and the epidermis was removed by dispase digestion. To allow cells to grow out from the tissue samples, the human dermis was placed in 6-well plates in DMEM-HG medium (Sigma Aldrich Co., cat. D6429) supplemented with 10% of FBS (fetal bovine serum, Sigma-Aldrich Co., F9665), 100 U/mL of penicillin and 100 μg/mL streptomycin, and placed under standard conditions (humidified atmosphere with 5% CO_2_ at 37 °C). The medium was changed every 2–3 days.

### Cell culture conditions

Human HaCaT keratinocytes (DKFZ, Heidelberg, Germany)^47,48^, the human dermal fibroblast 46BR.1N cell line (ECACC, Sigma Aldrich Co.) and human primary dermal fibroblasts used in this study were routinely cultured in culture flasks (growth surface area: 25 cm^2^) in DMEM-HG medium supplemented with 10% fetal bovine serum, 100 U/mL penicillin and 100 mg/ml streptomycin under standard conditions.

### XTT proliferation and LDH cytotoxicity assays

Briefly, the HaCaT and 46BR.1N cells were seeded in 96-well plates at a density of 5000 per well. Cell stimulation with RADA derivatives was performed using a protocol described before^49,50^. Both assays were performed according to the manufacturers’ instructions (XTT- Roche, Cat. No. 11465015001; LDH- Takara, Cat. No. MK401).

### Fibroblast viability assay

RADA peptides were dissolved in water at a concentration of 1.0%, placed in culture inserts (0.4 μm) in 24-well plates, and incubated for at least 1 h at 37 °C to allow gelation. DMEM-HG medium was then added at a volume double that of the hydrogel volume and the mixture was left overnight in an incubator (5% CO_2_ at 37 °C). Human primary dermal fibroblasts were seeded on the hydrogels, at a density of 50,000 per insert and in DMEM-HG medium with 2% FBS, and incubated under standard conditions. The medium was changed every 2 days. After 3 days of incubation, cells were stained with carboxyfluorescein succinimidyl ester (CFSE) (10 μM) and propidium iodide (100 µM) for 3 min. and then analyzed by fluorescence microscopy to assess their viability.

### Wound healing in vivo model

Eight to ten-week-old BALB/c female mice were used for these experiments. All experimental procedures were approved by the Local Ethics Committee for Animal Experimentation in Bydgoszcz, Poland (Approval No. 49/2016). The study was conducted in compliance with the ARRIVE gudidelines. All animal handling methods were carried out in accordance with European Union Commission Recommendation of 18 June 2007 on guidelines for the accommodation and care of animals used for experimental and other scientific purposes. Before experiments, mice were randomized and divided into groups comprising six individuals. The mice were anaesthetized with inhaled 2–5% isoflurane. The skin on the dorsum was shaved and disinfected. After the skin was folded and raised cranially and caudally along the spine, the mouse was placed in a lateral position, and the folded skin was pierced with a 6.0 mm biopsy punch to make two through-and-through symmetrical wounds. Next, 10 µL of peptide hybrid hydrogel was applied onto each wound. Every gel was prepared in sterile conditions in biological safety cabinet, using sterile water to final concentration of 10 mg/ml (1%). Then the gels were incubated for 24 h allowing it to assemble. Control mice were treated with RADA16-I. The wounds were covered with a transparent Tegaderm TM dressing to protect the wound from outer environment of the cage and other animals. It was fastened with an adhesive plaster wrapped around the animal’s torso. The peptide hybrids were applied daily to the wounds for five consecutive days. The dressings were replaced daily in the first five days of the experiment and every second day until the end of the second week of the experiment. To measure the wound areas the wounds were photographed with a ruler placed next to the injury site. Wound areas were calculated using the computer-assisted image analysis program ImageJ^51^.

### Tissue isolation for histological analyses

Mice were sacrificed on day 18 post-injury. In addition, for investigation of early wound healing, 1 mouse from each group was sacrificed on day 4 post-injury. The skin samples were collected and stored in formalin. Skin samples embedded in paraffin were cut into 5 µm sections following staining with Masson’s trichrome (green: collagen, dermis; grey or dark grey: cell nuclei, epidermis, sebaceous glands; dark red: keratinized epidermis, muscle, hair; light red: red blood cells). Image acquisitions were made with a Leica DM IL microscope.

### Quantification of collagen density

To evaluate the collagen density in dorsal skin wounds, we employed the method of colour deconvolution described by *Ruifrok and Johnston*^52^. Using the ImageJ software with a colour deconvolution plug-in^53^, we analyzed between 7 and 9 images of dorsal skin samples stained with Masson’ trichrome, representing 2–3 mice per group. The results are presented as mean relative collagen density.

### Statistical analysis

Statistical significance for the XTT and LDH experiments was determined with the non-parametric Mann–Whitney U test (*p* < 0.05) using Statistica 13.3 software (Statsoft, Warsaw, Poland). The graphs were prepared with GraphPad Prism 8 (GraphPad Software, La Jolla, CA, USA).

Statistical analysis of the data from the animal experiments was performed with a two-tailed Mann–Whitney U-test (*p* < 0.05) using XLSTAT (Addinsoft).

Statistically significant results were denoted as follows: *—*p* < 0.05 ; **—*p* < 0.01; ***—*p* < 0.001.

## Results

### Design and synthesis of peptide hybrids

The overall concept of these hybrids are shown in Fig. 1. A designed nanoformulation of hybrid gels is a multifunctional system that serves as a scaffold, facilitates the delivery of active compounds, and features a controlled release mechanism. Specifically, the RADA16-I peptide sequence provides scaffold functionality (fibril structure—blue color in Fig. 1), while active compounds are carried within the gel matrix. The controlled release mechanism allows for the triggered release of the active compounds (green color in Fig. 1) in response to a specific stimulus, which is typically associated with wound stress and involves the activity of elastase (orange color in Fig. 1).

In this work, the peptides were functionalized at their carboxyl-terminus with short sequences whose biological activity is well established. GHK, KGHK and RDKVYR have been proven to be factors which accelerate wound healing^38–40,42^. Peptide hybrids were designed to stimulate angiogenesis, attract immune and endothelial cells during injury and increase proliferation of fibroblasts while slowly releasing the signaling sequence. The triggering catalyst which releases the active sequence is human neutrophil elastase, which cuts the hybrid peptides within the AAPV site. It is worth emphasizing that neutrophilic elastase is present in wound, apart from MMP7 and MMP9, which are most prevalent in such locations, and, remarkably, it is upregulated in infected wound samples^54^. Therefore, when designing the amino acid sequence of the studied peptides, we chose a sequence that is specific to this enzyme.

All the designed scaffolds are based on the tetrapeptide sequence Arg-Ala-Asp-Ala repeated four times, a glycine linker, Ala-Ala-Pro-Val, another glycine linker and a functional motif, which are presented in a general scheme in Fig. 1. The linkers were added for better access for the enzyme to the cleavage site, as the RADA16-I structure is tightly arranged—a factor which is crucial for its gelation. The designed peptides are listed in Table 1.

**Table 1:** Peptide coding, their sequence, and theoretical and experimental masses.

Code	Sequence	Theoretical monoisotopic mass [Da]	Experimental monoisotopic mass [Da]
RADA16-I	Ac-(Arg-Ala-Asp-Ala)_4_-NH_2_	1711.846	1711.858
RADA-IM	Ac-(Arg-Ala-Asp-Ala)_4_-Gly-Gly-Gly-Ala-Ala-Pro-Val-Gly-Gly-Gly-Arg-Asp-Lys-Val-Tyr-Arg-NH_2_	3209.621	3209.562
RADA-GHK	Ac-(Arg-Ala-Asp-Ala)_4_-Gly-Gly-Gly-Ala-Ala-Pro-Val-Gly-Gly-Gly-His-Lys-NH_2_	2657.324	2657.260
RADA-KGHK	Ac-(Arg-Ala-Asp-Ala)_4_-Gly-Gly-Gly-Ala-Ala-Pro-Val-Gly-Gly-Gly-Lys-Gly-His-Lys-NH_2_	2842.441	2842.370

### Neutrophil elastase cleavage activity

**Figure 2:**
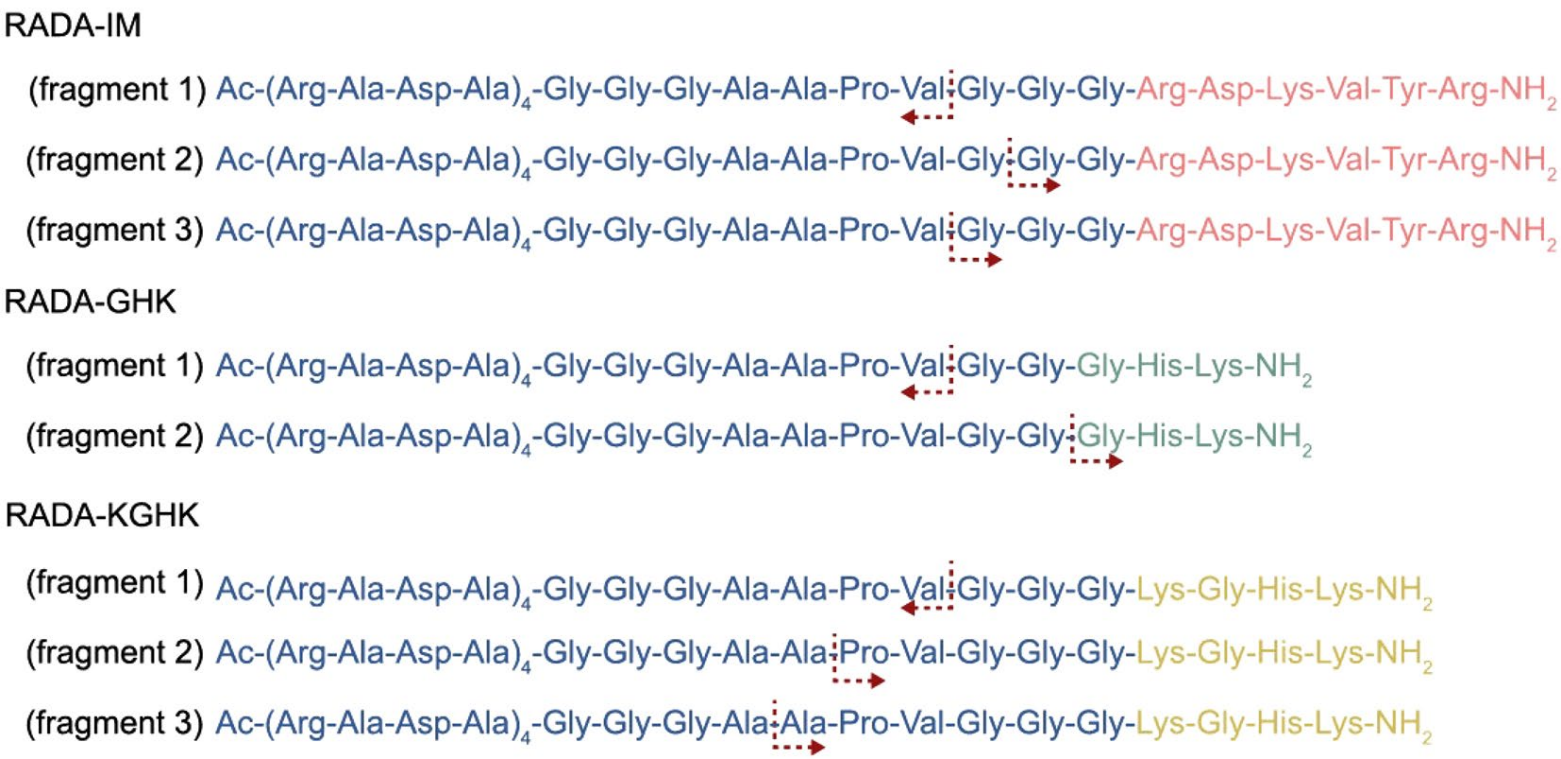
Elastase-digestion fragments of most abundant signals of peptide hybrids observed in mass spectrum analysis. The red arrows indicate the source of the identified degradation fragments.

The purpose behind this study is to use RADA hybrids to release small active peptides following elastase digestion at a wound site. To test if such a mechanism is feasible, the in vitro biostability of the peptides was investigated with neutrophil elastase treatment at physiological temperature. Digestion of the peptides was tracked by HPLC, and the released fragments were identified by mass spectrometry. The hybrids revealed the degradation profile presented in Fig. 2. The results show that the main fragments of RADA-IM elastase digestion were Gly^25^Gly^26^Arg^27^Asp^28^Lys^29^Val^30^Tyr^31^Arg^32^ and Gly^24^Gly^25^Gly^26^Arg^27^Asp^28^Lys^29^Val^30^Tyr^31^Arg^32^ (Fig. 2—RADA-IM—fragments 2 and 3). From the degradation of RADA-GHK, the predominantly observed fragment was Gly^26^His^27^Lys^28^ (Fig. 2—RADA-GHK—fragment 2). The prevailing fragment determined following RADA-KGHK degradation was Pro^22^Val^23^Gly^24^Gly^25^Gly^26^Lys^27^Gly^28^His^29^Lys^30^ and Ala^21^Pro^22^Val^23^Gly^24^Gly^25^Gly^26^Lys^27^Gly^28^His^29^Lys^30^ (Fig. 2—RADA-KGHK—fragment 2 and 3). The table with all identified fragments are in Supplementary Data (Table 3S).

### β-sheet structure formation studies

**Figure 3:**
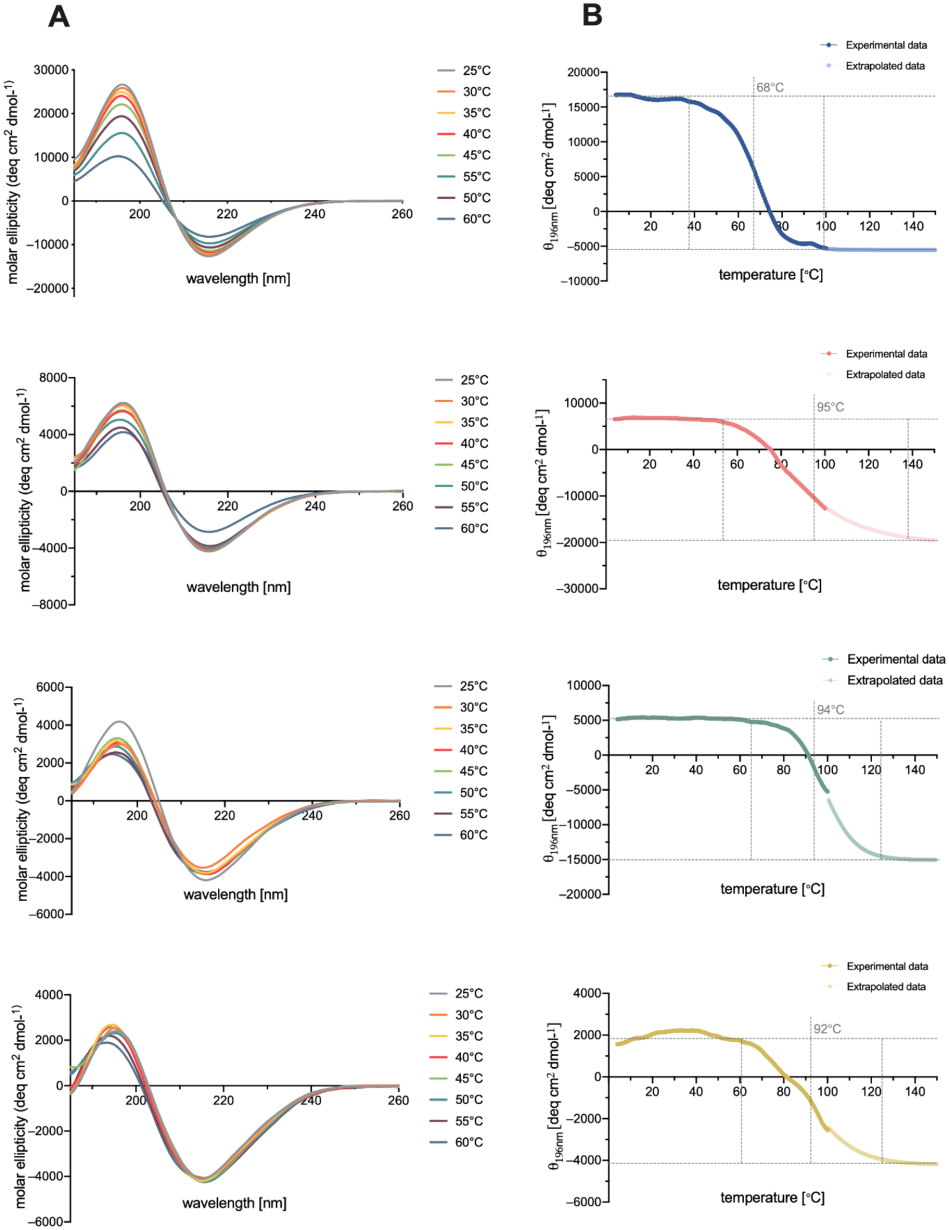
CD spectra of RADA peptides at 185–260 nm (**A**), and the effect of temperature on the CD spectra at constant 196 nm (**B**).

It has previously been established that various RADA16-I peptides assemble into stable β-sheet structures^13,25^. Circular dichroism was used as one of the tools for conformational examination of the studied peptide hybrids. The samples were incubated to form gels and then diluted to appropriate concentrations. All spectra of the synthesized peptides gave positive bands around 196 nm and a negative band at 218 nm, which indicated the presence of a β-sheet conformation, the same as in the original RADA16-I. However, the CD spectra show a decrease in the intensity of molar ellipticity between peptides (Fig. 3A), which means a lower ratio of β-sheet formation in the hybrid peptides. We subsequently examined the thermal stability of the RADA hybrids using CD. Figure 3B shows the CD spectra of peptides corresponding to mean molar ellipticities, [θ]_196_ nm, as a function of the temperature, over a range of 4–100 °C. Above 100 °C, the curve was extrapolated due to limitations caused by water, as above this temperature it would evaporate and the concentration of peptide would drastically change. The first disruption of the β-sheets were observed around 38 °C for RADA, 52 °C for RADA-IM, 61 °C for RADA-KGHK, and 65 °C for RADA-GHK, as indicated by decreases of molar ellipticity. The transition temperatures, representing the state in which half of the structure forms a β-conformation and half is disordered, were 68 °C for RADA, 95 °C for RADA-IM, 94 °C for RADA-GHK, and about 92 °C for RADA-KGHK.

**Figure 4:**
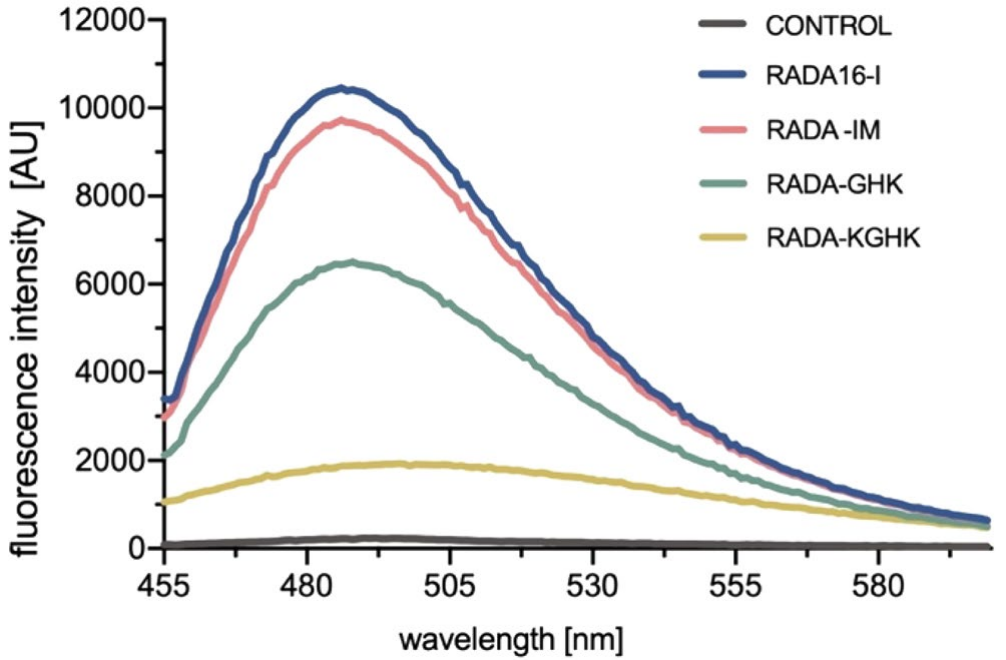
Thioflavin T test for RADA peptide hybrids.

In the next step, analysis was carried out to confirm the presence of β-structures that form a complex with thioflavin T (ThT). This technique, which is used to detect amyloid fibrils, has successfully been applied to image RADA16-I nanofibers^55^. Figure 4 demonstrates that thioflavin T formed a complex with the studied RADA fibres, which was indicated by fluorescence intensity at 482 nm. The differences between the fluorescence intensities reflected different ratios of formation of β-sheets.

The peptide elongated with KGHK showed a lower potential for β-sheet formation in comparison to RADA16-I itself. Interestingly, RADA-IM, a peptide longer than RADA-KGHK by only two amino acid residues, has a similar β-sheet formation potential to RADA16-I (Fig. 4).

### Nanofibre structure formation

**Figure 5:**
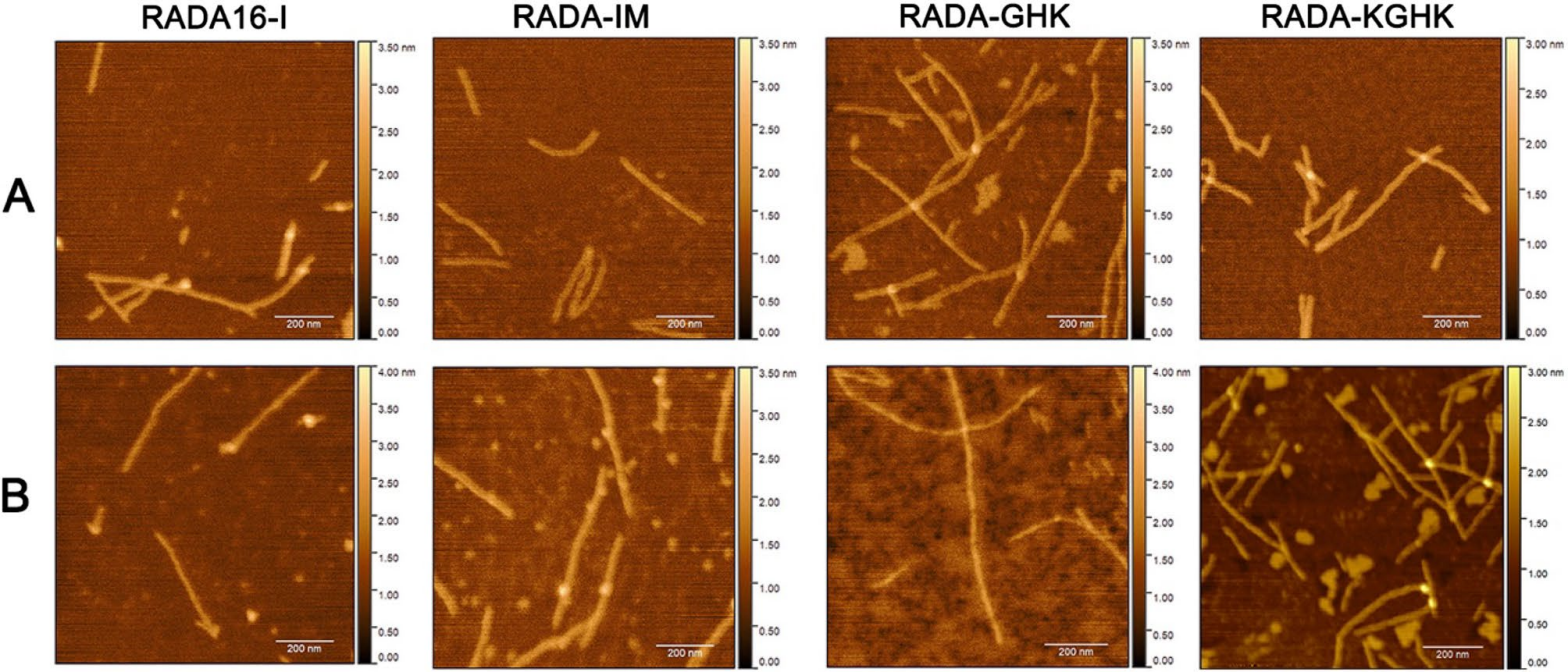
AFM images of RADA16-I and RADA hybrids after incubation in aqueous solution (**A**), and after sonication and reassembly over 24 h (**B**).

In order to characterize nanofibre formation we used a combination of AFM and TEM, and to determine gel morphology we used cryoSEM. The peptide fibres formed by RADA16-I, RADA-IM, RADA-GHK and RADA-KGHK have a similar shape visible in topographic images. The fiber length estimated from AFM images ranges between 100 and 800 nm, with a mean height of 5–6 nm. Selected topographic images are presented in Fig. 5A. In the next step those samples with formed fibres were sonicated to cause disintegration into smaller particles (Fig. 2S), then incubated at 37 °C for the next 24 h. All hybrids showed the same properties of reassembly as the original length fibres, without compromising the original concept of the hydrogel (Fig. 5B), and demonstrated an ability to reassemble in a short time.

**Figure 6:**
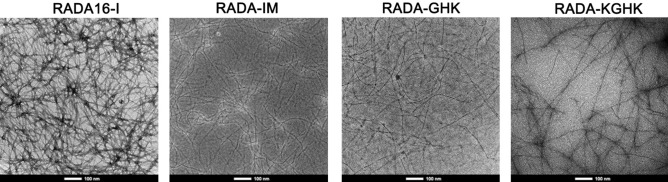
TEM images of RADA16-I and RADA hybrids.

Similarly, TEM images showed a dense interwoven and tangled network of fibres for all the peptide hybrids (Fig. 6). RADA16-I fibres seemed to be straight, whereas RADA-IM, RADA-GHK and RADA-KGHK fibres appeared thinner and more delicate. All fibres had significant lengths varying between 100 and 600 nm. RADA16-I itself created fibres of 15–20 nm thickness, whereas RADA-IM fibres were 6–8 nm thick and RADA-GHK and RADA-KGHK fibres were about 3 nm thick. However, TEM and AFM results confirmed the potential use of these hybrids as scaffolds.

### Hydrogel morphology

**Figure 7:**
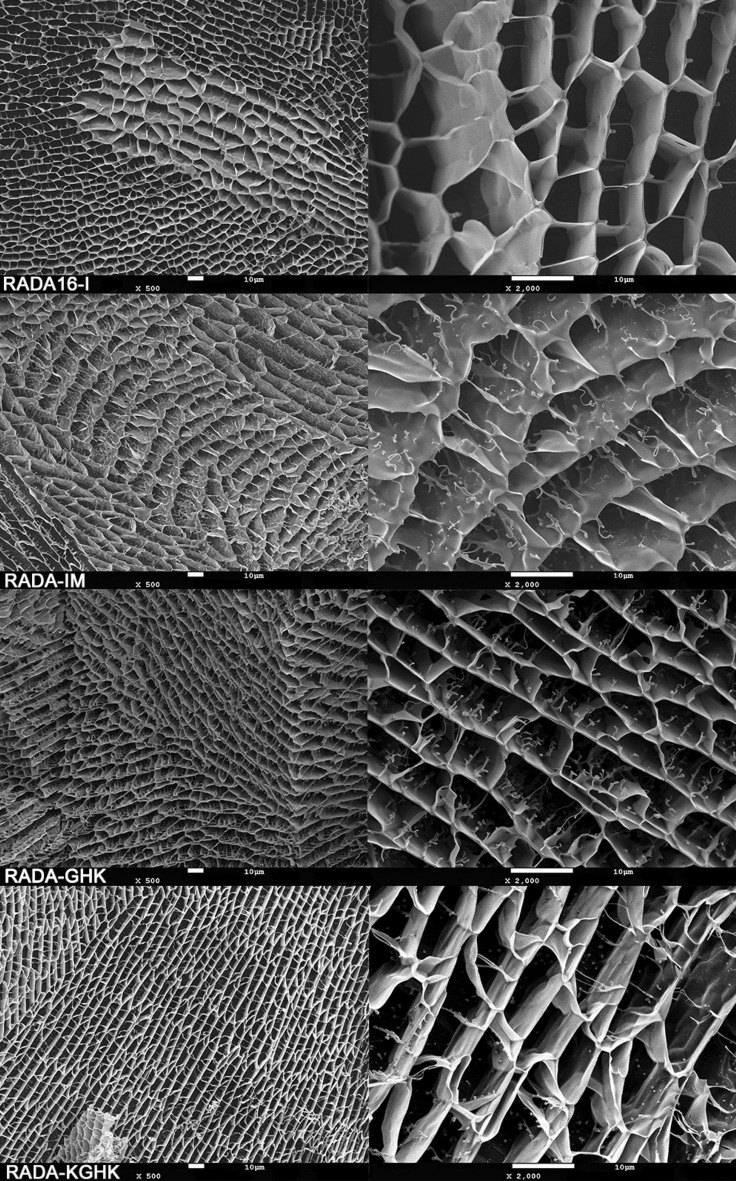
CryoSEM images of RADA peptides. The photographs represent a surface view of the hydrogel.

In the next stage of research we checked the morphology of the studied hydrogels. For this purpose, the gels were frozen rapidly in liquid nitrogen (− 210 °C), and then water was partially sublimated. The images showed that all peptides were highly organized, resembling a honeycomb (Fig. 7). The morphology was porous and had apparently interconnected pores, and consisted of layers with spaces about 6–8 µm in height. The spaces were separated by thin membranous walls less than 0.1 µm thick. In the magnification, it can be seen that the modified peptides had characteristic branches inside this honeycomb-like structure, while RADA16-I did not.

### Hydrogel properties

**Figure 8:**
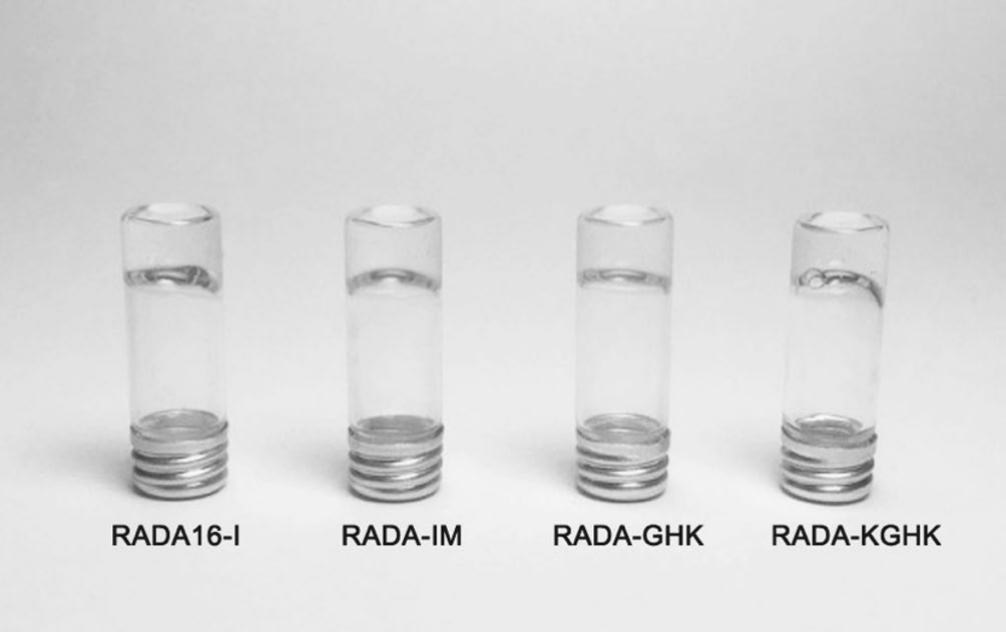
Images of RADA peptide hybrids after the gelling process.

Peptide solutions containing water were prepared at a concentration of 10 mg/ml (1%, v/v). The pH was adjusted to 7.4 with KOH and the solutions were incubated at 37 °C. Hybrids formed soft and transparent gels, which visually showed no difference from the original RADA16-I hydrogel, despite being 12–17 amino acids longer (Fig. 8).

**Figure 9:**
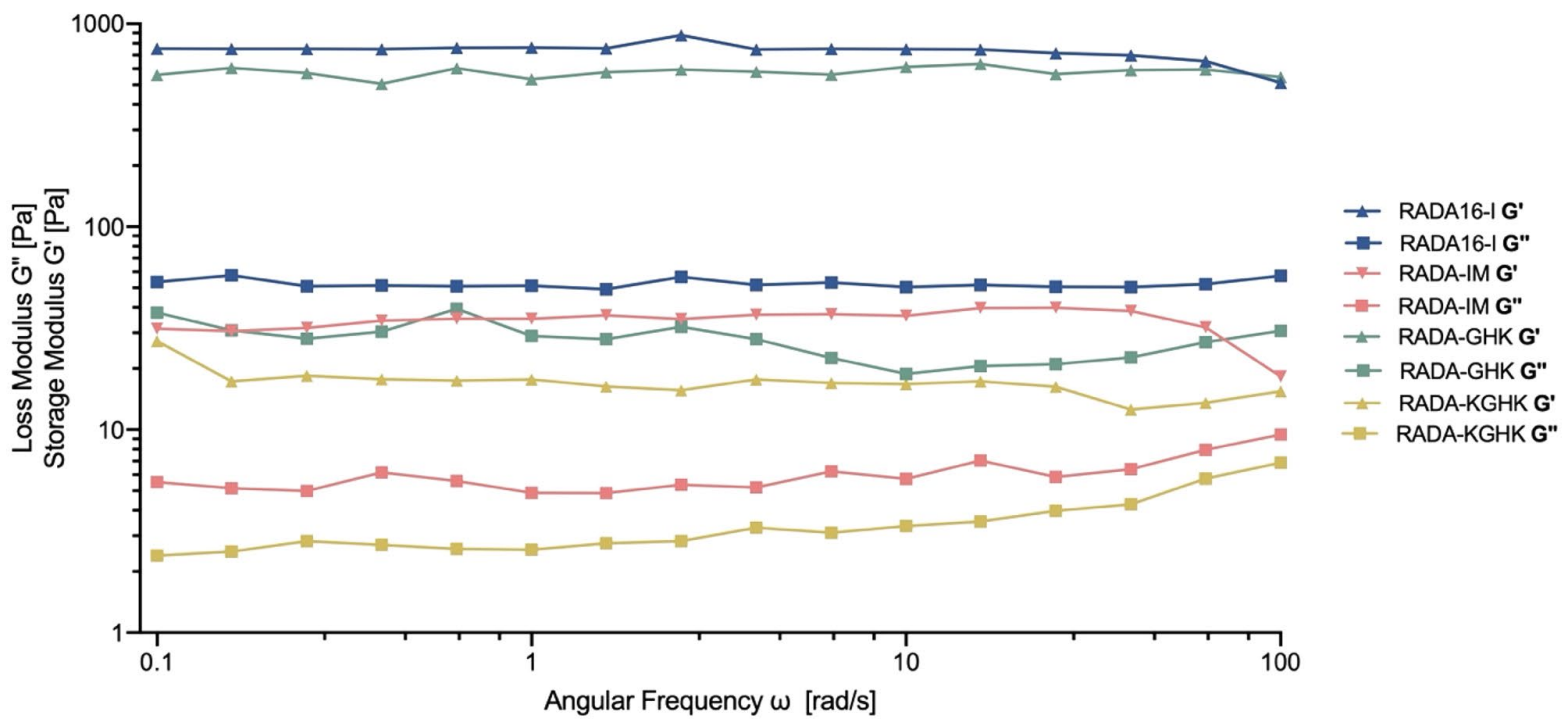
Mechanical characterization of hybrid peptides by dynamic frequency sweep test, at 1% concentration, ϒ = 1%, 37 °C.

The viscoelastic properties of the hybrids were evaluated at physiological temperature (37 °C) by dynamic rheometry using cone-plate geometry. For all the studied gels the storage modulus (G’) was greater than the loss modulus G”, and both were relatively independent of the oscillatory frequency applied, indicating a gel’s elastic profile (Fig. 9). The storage modulus G’ reached a value of 550 Pa for the RADA-GHK peptide (Fig. 9), almost the same as for the original RADA16-I peptide. The magnitude of G’ for the peptides RADA-IM and RADA-KGHK was around one-tenth of these values. (Fig. 9).

**Figure 10:**
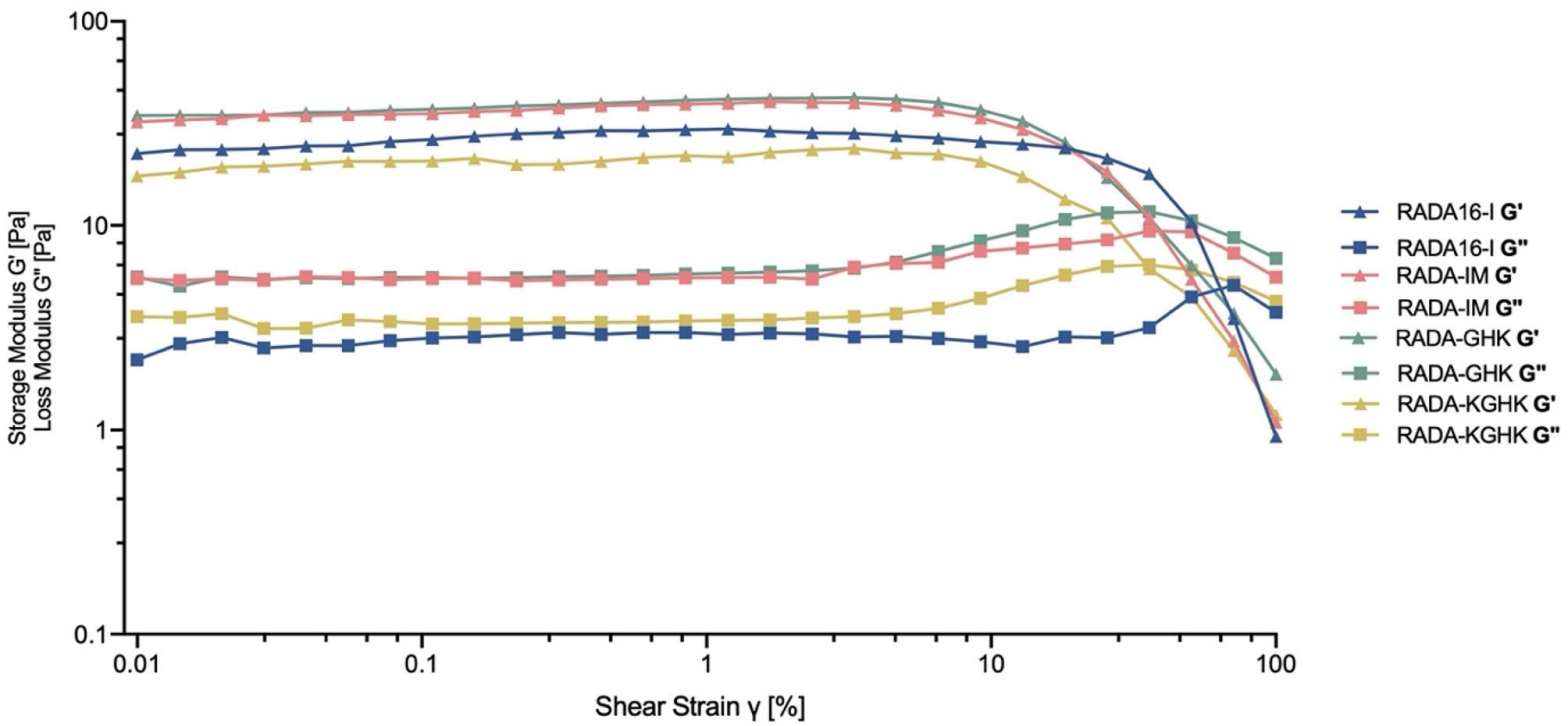
G’ and G” of RADA16-I hybrids as a function of shear strain at ω = 6.28 rad/s and 37 °C.

All hybrids showed good shear-thinning properties. Strain sweep curves of hybrids remained constant in the low shear strain region (strain < 10%), however they indicated a rapid decay in G’ above 10% strain and, ultimately, behaviour as a liquid (G” > G’) as the shear strain percentage value progressed. RADA16-I behaved like a liquid above 40%, RADA-GHK and RADA-IM above 30% and RADA-KGHK above 25% of shear strain (Fig. 10). All the hybrids exhibited elasticity at rest and up to 4 Pa; above this value, they behaved like liquids (Fig. 3S). This property is necessary to keep cells in suspension in the gel. The application of shear stress resulted in a decrease in these properties, presumably due to disruption of the hydrogen bonds between amino acids in the RADA16-I chains.

### Stability in water and presence of free peptide in human plasma

During incubation for 24 h in water there was, at every time point, no reduction of signal for the peptides (Fig. 4S). It can thus be concluded that the peptides are stable in aqueous solutions. The presence of RADA16-I and the RADA hybrids in human plasma, over time, was also studied. The HPLC signal for peptides without any chromophores and with a complexed matrix can be hard to detect at UV–Vis wavelengths; that is why some time points were additionally measured by mass spectrometry. The chromatogram of the samples at the 0 min time point showed almost no signal of peptide. This sample goes through the same procedure as all incubated samples, including removing albumins by ethanol precipitation, excluding the incubation at 37 °C. Due to that procedure, if peptides bind to albumins, they will be removed in that manner. In order to establish potential binding to albumins, we performed tests to determine affinity of the hybrid peptides to albumins. The process was followed by mass spectrometry, and the MS spectrum of the supernatant fraction and of the last wash fraction is shown in Fig. 5S of the Supplementary Data. It can be speculated that all the peptides interacted with bovine albumin since the m/z peak in the elution fraction was consistent with the mass of the peptides.

### XTT proliferation and LDH cytotoxicity assays

**Figure 11:**
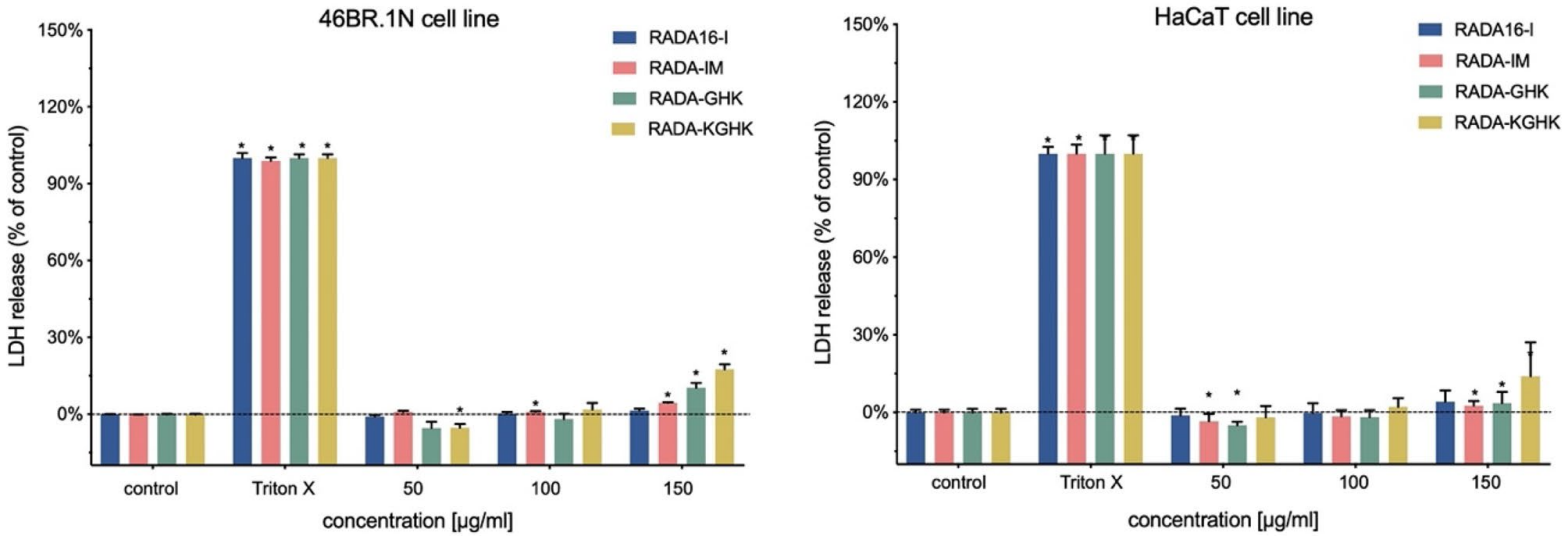
Cytotoxicity of RADA hybrids towards 46BR.1N fibroblasts and HaCaT keratinocytes after 48 h of incubation. The graph shows the results from 3 independent experiments (4 replicates in each, n = 12). The results are presented as mean with SEM. Statistically significant differences compared to control were determined with the Mann–Whitney U test at *p*-value < 0.05 and are indicated with an asterisk “*”. TRITON X- positive control- cells grown in a medium containing 1% TRITON X, where maximum LDH release is observed, indicates maximum cytotoxicity.

Assessment of the potential cytotoxicity of a peptide is a much-needed experiment for every new compound before beginning in vivo studies. The studied concentrations do not undergo a gelation process. An LDH test showed that the studied peptides were not cytotoxic to human skin cells (Fig. 11). A slight effect (ca. 15%) was seen at a concentration of 150 µg/ml for RADA-KGHK for both cell lines and for RADA-GHK in 46BR.1N fibroblasts.

**Figure 12:**
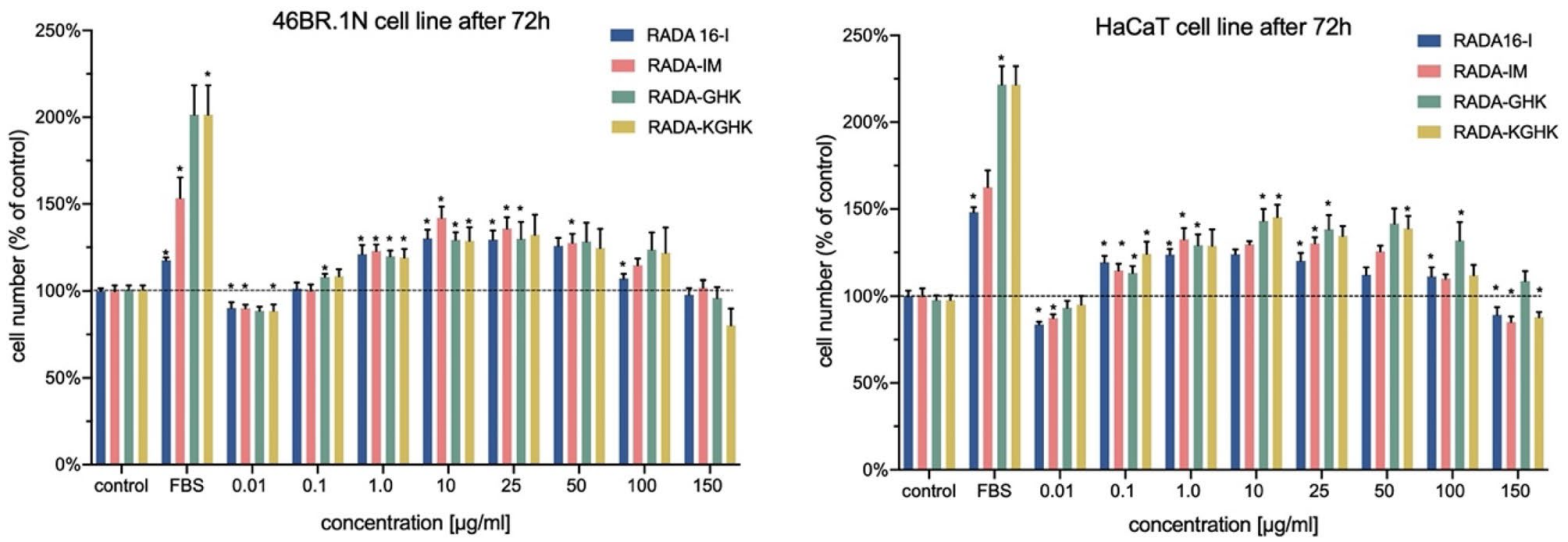
RADA hybrid proliferation test for 46BR.1N fibroblasts and HaCaT keratinocytes after 72 h of incubation. The histogram bars represent results of 3 independent experiments (4 replicates in each, n = 12), and the error bars represent SEM. Statistically significant differences compared to control were determined with the Mann–Whitney U test at *p*-value < 0.05 and are indicated with an asterisk “*”. FBS—positive control—cells grown in medium containing 10% FBS.

The results obtained in the XTT analysis of the effects of the peptides on proliferation of human 46BR.1N fibroblasts and HaCaT keratinocytes (Fig. 12) showed that the peptides stimulated the proliferation of human skin cells. The most prominent pro-proliferating effect can be observed at concentrations of 1–50 ug/ml. This effect was slightly stronger for the HaCaT keratinocytes cell line, whose proliferation was increased by 30–45% compared to the control at concentrations of 10–50 µg/ml. In both cases, the pro-proliferating effect was more potent after 72 h of incubation (48 h incubation graph can be found in Suplementary Data Fig. 6S), which was consistent with long-term application. In addition, the peptides did not cause inhibition of proliferation of both tested cell lines.

### Fibroblast viability assay

**Figure 13:**
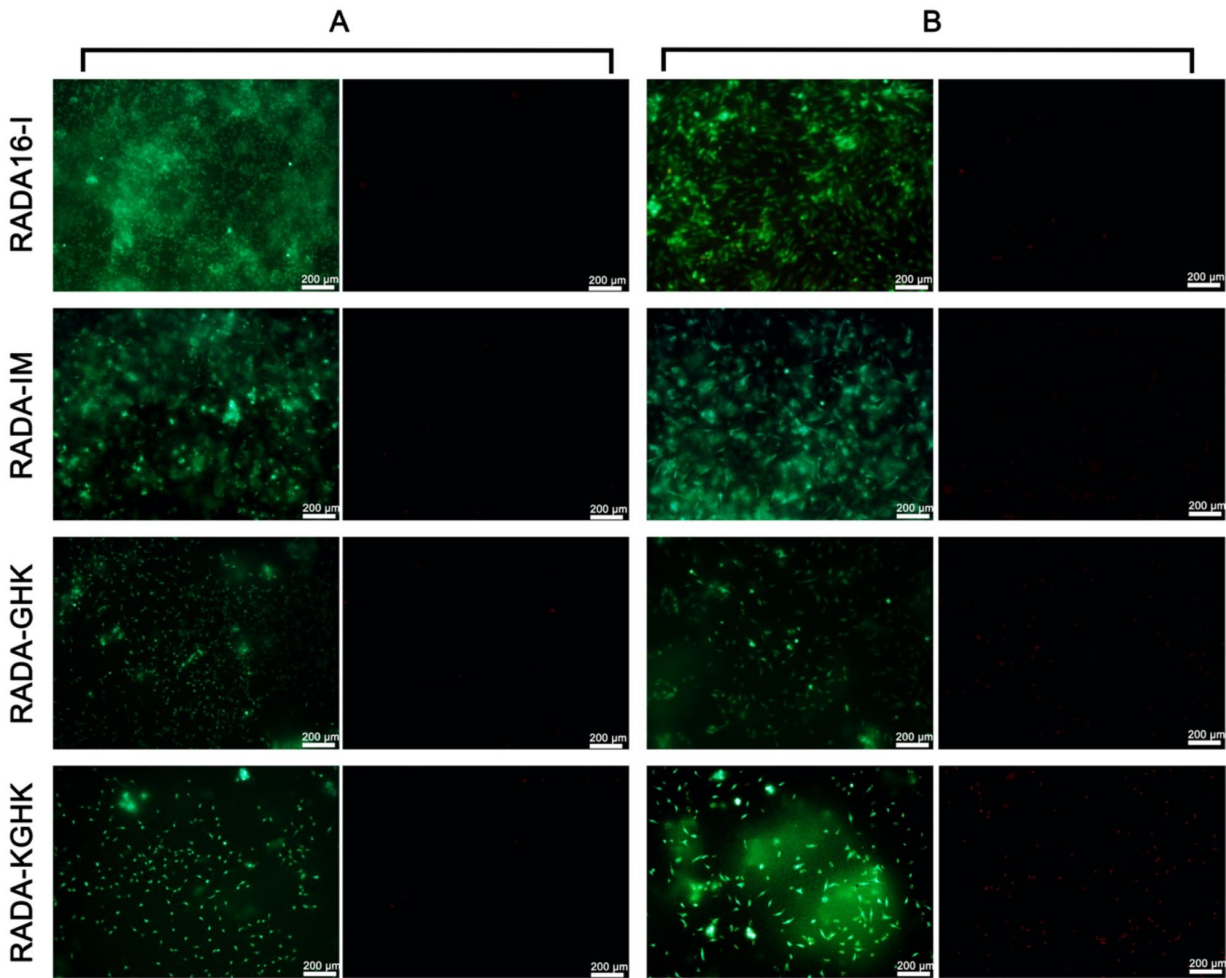
Microscope images of 46BR.1N fibroblasts (**A**) and primary fibroblasts (**B**) after seeding on RADA hybrids for 72 h.

The original RADA16-I peptide hydrogel is transparent, as are those of the hybrids. This feature is essential for accurate image collection during 3D-cell and tissue cultures. The level of cytotoxicity can be tested not only by LDH but also by viability tests. Therefore, we tested the viability of human dermal fibroblasts (46BR.1N cell line and primary cells expanded from human dermis) in the studied scaffolds in this experiment. The human cells were seeded on 1% hydrogels after exchange of water for DMEM-HG medium. A similar procedure has previously been used by *Kumada et al.*^35^. After three days of incubation, cell viability was observed under a fluorescence microscope (Fig. 13). Both types of cells remained viable (Fig. 13A,B; green staining, left panels) on the tested scaffolds after 3 days of incubation. Only single dead 46BR.1N cells were observed on all the tested scaffolds, as demonstrated by staining with propidium iodide (Fig. 13A; right panels). For primary dermal fibroblasts, most dead cells were observed after incubation on the RADA-KGHK scaffold (Fig. 13B; right panels). Primary fibroblasts showed a characteristic spindle-shaped morphology when incubated on all the tested RADA hydrogels and 46BR.1N cells also retained normal morphology.

### Skin wound healing model in mice

As cytotoxicity, proliferation assay and viability tests proved the safety and biocompatibility of RADA hybrids, we decided to conduct in vivo experiments in a skin wound healing model. We tested a topical administration of hydrogels in cutaneous full-thickness excisional wounds in mice, an often-used model to test pharmacological solutions and dressings for skin wound healing^56^.

**Figure 14:**
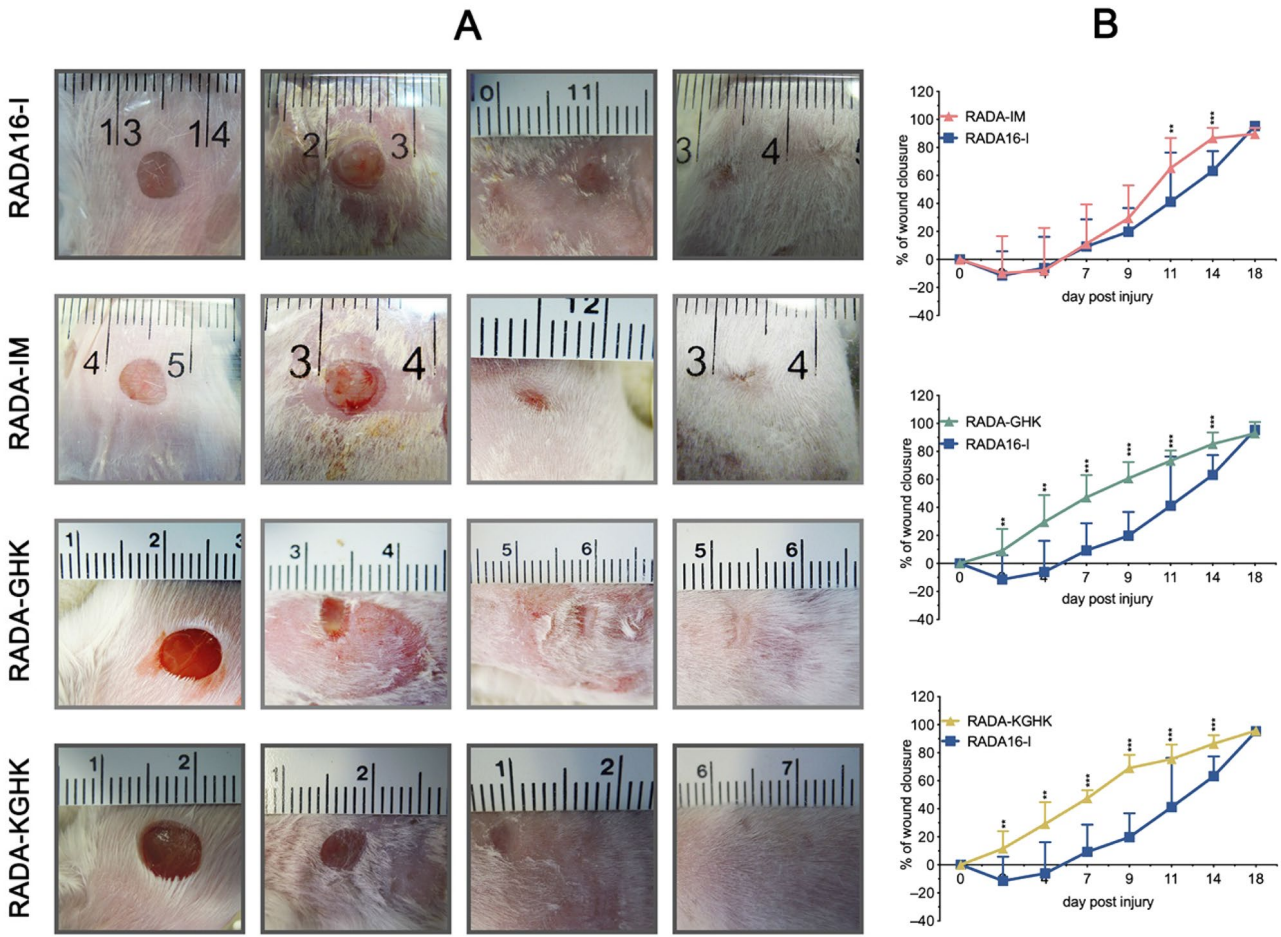
Skin healing process in wounds treated with RADA hybrid hydrogels contrasted with saline-treated controls. (**A**) representative photographs of the wound healing process on days 0, 7, 14, and 18 post-injury; (**B**) the mean area of wounds throughout the experiment; n = 6; error bars represent SD. Statistically significant differences compared to control were determined with the Mann–Whitney U test at *p*-value < 0.05. Significant *p*-values < 0.05, < 0.05, and 0.001 are marked with single “*”, double “**”, and triple asterisks “***”, respectively.

In this study, full-thickness, excisional wounds were made on the dorsum of BALB/c mice with a biopsy punch. The peptide hybrids (at a concentration of 1%) were applied topically. The control group was treated with RADA16-I hydrogel containing no modifications. In addition, four mice were euthanized on day four to examine the early stage of wound healing. The progress of wound healing was evaluated based on photographic documentation collected during the experiment (Fig. 14A,B). Accelerated wound healing compared to RADA16-I was observed following application of the RADA-GHK and RADA-KGHK peptide hybrids. In mice treated with these hybrids wound closure began faster and was significantly and consistently accelerated from day 2 up to day 14, compared to the groups treated with RADA16-I or RADA-IM (Fig. 14B). On day 7 of the experiment, the groups treated with the RADA-GHK and the RADA-KGHK showed more than 40% wound closure, specifically 41.8 ± 16.1% and 44.4 ± 4.9% respectively. In contrast, at that time, the groups treated with RADA16-I and RADA-IM showed minimal improvement to wound closure, specifically 11.1 ± 21%, and 16.2 ± 21.1% respectively. On day 18, complete skin wound closure was achieved for all the tested peptides.

### Histological evaluation of skin samples

**Figure 15:**
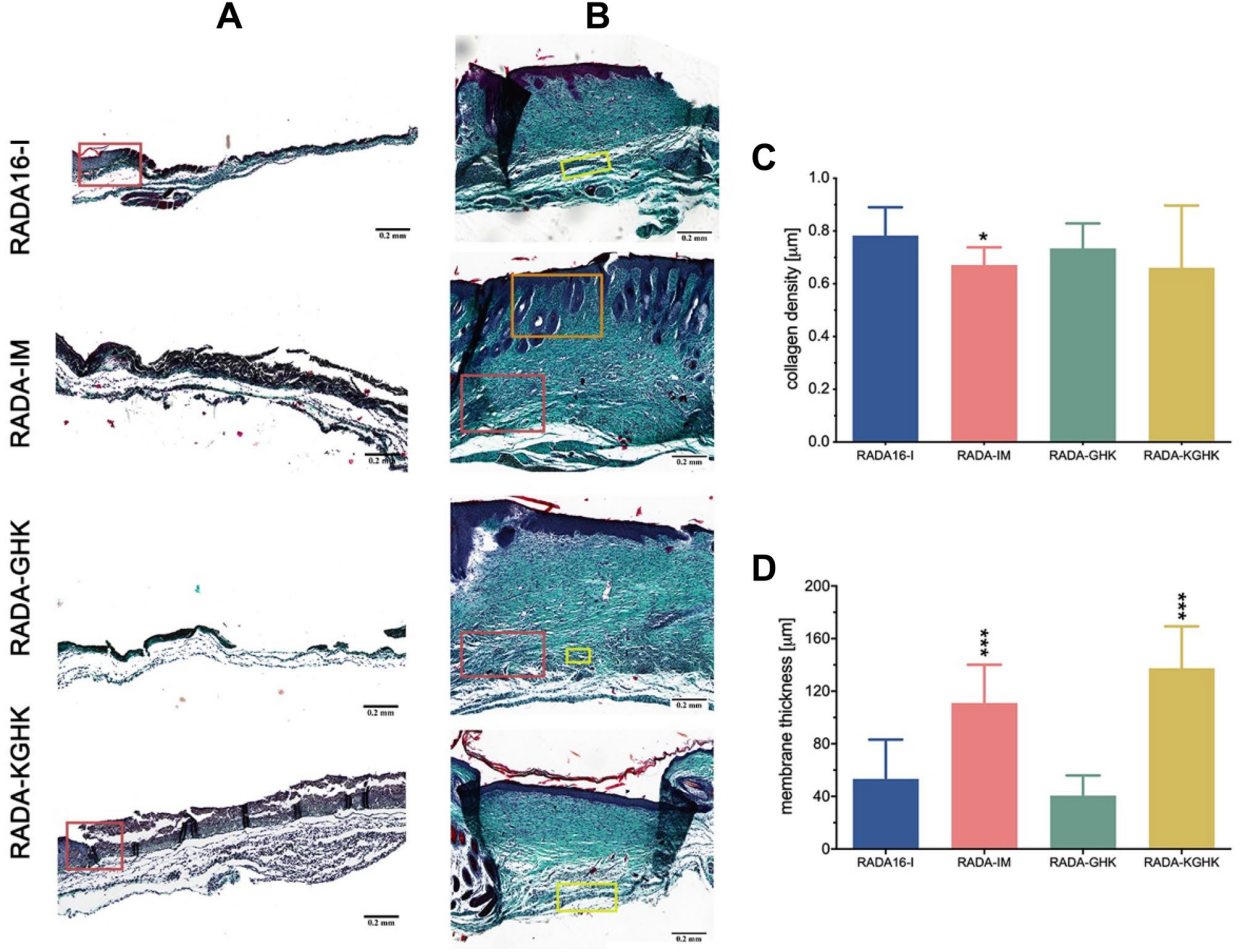
Images of dorsal skin samples stained with Masson’s trichrome stain (**A**), (**B**). (**A**) Samples collected on day 4 after injury show significant differences in tissue thickness, with a membrane-like structure (epithelium) covering the wound and keratinocytes visible on the edge of the peptide-treated wounds (brown square). (**B**) Skin samples of tissue treated with RADA16-I, RADA-GHK or RADA-KGHK harvested on day 18 show significant differences in tissue architecture to skin stimulated with RADA-IM peptide, where more hair follicles could be observed. Hair follicles (orange square), muscle-like cells (red square) and nuclei-dense regions (yellow square) can be observed in the latter tissues. (**C**) Skin treated with RADA-IM shows statistically significant lower collagen density than the RADA16-I sample on day 18 post-injury. (**D**) Quantification of epithelium thickness on day 4 post-injury shows that wounds treated with peptide RADA-IM and RADA-KGHK hybrids have significantly thicker epithelium than those of the RADA16-I group. Statistical significance was determined with the Mann–Whitney U test; one (*), two (**), or three asterisks (***) indicate *p* < 0 .05, *p* < 0 .01, or *p* < 0 .001, respectively.

Within two days after inflicting injury, a membrane-like structure had formed in the wound area. This structure did not appear to be epithelial in nature and formed on the basis of the wound. One or two mice from each group were sacrificed on day 4, and their dorsal skin was sampled to examine the phenomenon (Fig. 15A). The membrane-like structure consisted mainly of fibroblasts with occasional keratinocytes found on the wound’s edges in skin treated with RADA16-I, RADA-IM, RADA-GHK, and RADA-KGHK. All the applied hybrid peptides significantly increased the thickness of the membrane in comparison to RADA16-I. Statistical analysis proved the differences between the control treated with RADA16-I and those treated with peptide-hybrids to be significant (*p*-value < 0.001), except for skin treated with RADA-GHK (Fig. 15D). The RADA-IM and RADA-KGHK peptides promoted formation of the membrane-like structure most effectively.

To evaluate the quality of the restored tissue, dorsal skin around the wound area was harvested on day 18 after injury and stained by Masson’s method. On day 18, the surfaces of all wounds dressed with peptides were covered entirely with epidermis, as shown in Fig. 15B. All dressed wounds appeared to have well-developed epidermis containing multilayered keratinocytes with keratin resembling intact normal skin. The tissues beneath the wound surfaces were found to be compact and strong. Although the wound surfaces were covered with a new epidermis comprising keratin, the formation of hair follicles and sebaceous glands was only sparsely observed, except for the skin dressed with RADA-IM hydrogel. In all the studied samples the basal layer of cells in the wound area showed a high density of cell nuclei over the collagenous extracellular matrix. In all groups, a number of cells similar in morphology to the muscle cells of panniculus carnosus are visible. This suggests ongoing restoration of the muscle layer of the skin. In the skin samples treated with RADA-IM, many hair follicles could be observed, not only in the epidermis but also in the basal layer of the wound area. In contrast, in the other peptide-treated groups, hair follicles are sporadically observed on the fringes of the wound area. The histological image of the skin stimulated with RADA-IM hydrogel is most similar to the histological image of healthy skin (Fig. 7S) and we conclude that, of all the tested hydrogels, the RADA-IM gel stimulates the regeneration process most effectively. The analysis revealed an increased collagen density in the wounds following the application of the tested RADA peptides (Fig. 15C).

## Discussion

In this study, the idea was to combine the properties of hydrogel scaffolds with biologically active small peptides. RADA16-I hydrogels can be directly mixed with an active compound or can be easily functionalized by covalent bonding. In the first case, the hydrogel will release the compound slowly under aqueous conditions^57–59^; in the second case, an external biocatalyst is needed to release the active compound to the environment. An active compound co-formulated with a hydrogel can be released as the result of, e.g., accidental stirring and, in consequence, undergo immediate clearance. Covalent bonding lowers the release rate, despite mechanical mixing. The triggering sequence releasing the active peptide in this study is AAPV. This is a sequence which is susceptible to human neutrophil elastase, and it was chosen because that enzyme is one of the most abundant proteinases in the environment of chronic wounds^41^. During inflammation, one of the cellular responses to wounding, neutrophil recruitment takes place^60^. Two of the main tasks of neutrophils are phagocytosis and secretion of proteases, such as elastase^41,61^. In the presence of enzymes secreted into the wound site, the peptides used in this study slowly release the active compounds. Two of the selected active peptide sequences, GHK and KGHK, come from natural proteolysis of osteonectin (SPARC) and correspond to its amino acid sequence fragments 119–122 and 120–122, respectively^62^. Both these peptides stimulate angiogenesis. GHK binds copper, which makes it a mitogen/morphogen, although copper complexing is not required for stimulatory activity^62^. Another source of GHK peptides is degraded collagen which is hydrolyzed following injury by metalloproteinases secreted into the wound environment^63^. At the same time, GHK is a modulator of expression of MMP-1, MMP-2 and MMP-9 and their inhibitors, TIMP-1 and TIMP-2^64,65^. This activity accelerates wound healing processes and facilitates skin remodelling^66^. These processes include attracting immune and endothelial cells following injury^38^, decreasing inflammatory cytokine TNF-α levels^65^, improving nerve regeneration^67^, and increasing the proliferation of fibroblasts^43^.

RDKVYR^68^, is a modified peptide sequence derived from the human thymopoietin hormone (TH5) corresponding to amino acids 32–37. We recently proved that wounds treated with this peptide showed accelerated migration of keratinocytes and appeared to be richer in fibroblasts. In addition, it is involved in transcriptional induction of the *POUF5F1* gene encoding OCT4 pluripotency factor and the *TET1* and *TET3* genes involved in active DNA demethylation^40^. Both *TET* genes and *POUF5F1* are involved in skin regeneration processes.

We proved that the synthesized peptides released the active motifs when treated with elastase, as designed. Mass spectrometry analysis confirmed that all hybrid peptides were cleaved between Val and Gly immediately after the AAPV sequence (Fig. 2—fragments 1). In the case of RADA-IM, the digestion occurred after Gly, leaving the GGRDKVYR and GGGRDKVYR motif free. Some other fragments appeared on the RADA-KGHK mass spectrum, showing an additional aminoacids in cleaved fragmets ei. PV and APV, which is consistent with character of elastase cleaving peptides preferentially at the *C*-teminus of aliphatic aminoacids. Nevertheless, in all cases, fragments corresponding to the gelling fragment and the enzyme were identified. This concept, where an enzyme behaves as a triggering factor to release active motifs, has previously been reported^69,70^.

The CD spectra of the peptide hybrids revealed structural properties similar to those of RADA16-I. However, the hybrids showed a reduced β-sheet content. The Thioflavin T assay strongly supports the results from the CD studies. Molar ellipticity was most intense for RADA16-I, as was as the intensity of the emission spectra of ThT. The least abundant was RADA-KGHK. These observations would be in agreement with those for other modified RADA16-I peptides, no matter if the modifications were on the *N*-terminus^37^ or *C*-terminus^71,72^. It was expected that elongation of the RADA16-I sequence will definitely influence on its conformation. RADA-IM is longer by 16 aminoacids, where RADA16-I itself has 16 aminoacids in sequence. The decreased β-sheet formation of the hybrid peptides can be explained by steric hindrance resulting from the extended length compared to the RADA16-I peptide, as well as to the introduction of the unstructured and flexible fragment containing six glycine residues. We confirmed this phenomenon in our recent publication^73^ in which it can be seen that elongation of the RADA-I peptide sequence, significantly increases the disordered or α-helical structure compared to the dominant β-sheet structure for RADA16-I. However, it cannot be stated that the β-sheet formation ratio always decreases with peptide length. RADA-IM is longer by two amino acid residues in comparison to RADA-KGHK, yet despite that fact the intensities of the CD and ThT spectra are more abundant for the former than for the latter.

During the performance of CD scans at different temperatures we noticed that, in the case of RADA16-I, the intensity of signals is strongly dependent on temperature, which had previously been indicated for the original peptide and its variations^74–76^. Surprisingly, the β-sheets in our peptide hybrids are more stable. The disruption of the RADA16-I β-sheet structure begins at 45 °C, whereas the corresponding temperatures for the peptide hybrids are much higher. It is easily observed that the elongation of the RADA16-I sequence with additional amino acids resulted in an increase in heat stability. Among the designed hybrids, RADA16-I displays the lowest stability of β-sheet structure, whereas RADA-GHK has the highest. It is noteworthy that our calculations with the UNRES Coarse-Grained Model^73^ also show that at lower temperatures the peptides studied have a dominant β-structure while at higher thematatures the disordered or α-helical structure dominates. In addition, the modified peptides have greater thermal stability than the unmodified RADA16-I peptide. But, it cannot be generally stated that all RADA16-I modifications are more stable, because other articles report modified peptides that are less or more stable than the original peptide^77^. A possible cause may be the presence of extra positively charged residues in the hybrids, in comparison with RADA16-I, which may be an additional factor in their higher thermal stability. Proteins with a high content of charged amino acid residues are also characterized by high melting points^78,79^.

The next step was to evaluate if the newly designed RADA peptides are organized in the same way as the original RADA16-I, using AFM and TEM techniques. It was previously proven that RADA16-I forms highly organized nanofibers ranging from a few hundred nanometers up to a few microns in length^24^. Our peptides reassembled in the same way as the original RADA16-I, so it can be concluded that they maintained its reassembly properties, despite being elongated by 12 to 16 amino acids. Previously reported peptide hybrids tend to have the same length of fibres if appended with a short sequence (4 aa)^80^, and shorter fibres in comparison to the original peptide if appended by 12 aa^81^, or there may be no significant change at all despite an additional 7, 9 or 14 amino acids^37^. Some of the reported peptides were simply mixed with RADA16-I to obtain a hydrogel, which made the fibres identical to the original peptide^82^. TEM observations showed that our modified peptides are thinner, especially RADA-KGHK, however that does not make the biomaterial less useful as a scaffold. It was previously observed that thinner fibres are formed at lower concentrations of RADA16-I^74^. However, drastic differences in morphology of RADA16-I constructs with an additional six-aa sequence (GGGPQG) have been reported^70^. In this case, we can conclude that steric hindrance of the additional sequence may prevent nanofibre formation to some extent, which comes down to a lower concentration of molecules prone to assembly.

It is very interesting that the length of the fibrils obtained in these tests (100–600 nm) is comparable to the length of type 1 skin collagen fibrils (monomers are about 280–300 nm long)^83^. On the other hand, the diameter of peptide fibrils studied in this work (3–20 nm) is slightly larger than the diameter of a single type I collagen molecule (1.5 nm) but very similar to the diameter of collagen microfibrils present in the skin. Five monomers assemble into one microfibril, which is about 4 nm in diameter. The diameter of a mature collagen fibril is about 30–1000 nm^83^ and depends on the degree of its maturity, the type of collagen it forms and the tissue in which it occurs. Many microfibrils may associate to form collagen fibrils, and in skin the diameter of these fibrils can range from 30 to 300 nm.

Obtaining AFM and TEM images of peptide fibres required dilution of the hydrogels. The cryoSEM technique provides a possibility to see the undiluted microstructures of the hydrogels. Previously registered cryoSEM images of RADA16-I were acquired from an aqueous dispersion of lyophilized powder^84^. This technique revealed the porous morphology of the hybrids. This observation is in agreement with many other reported hydrogels, such as cellulosic super absorbent^85^, freeze-dried agarose scaffolds^86^, or collagen/chitosan scaffolds^87^. No alveolar pattern was observed, which is typical for ice artefacts in cryoSEM^88^. To our knowledge, these images are the first registered cryoSEM pictures for the RADA16-I hydrogel. The photographs of the RADA hybrids differ from unmodified RADA16-I in their characteristic branches inside the structures. These differences in morphology may be explained by parts of hydrogel not being cross-linked by normal interactions present in RADA peptides. It is probable that IM, GHK and KGHK, along with the glycine linkers and enzymatic sequence fragments, caused chunks of unfolded peptides and the occurrence of these ramifications. To prove that, there is a need to analyze differently modified RADA16-I peptides which can form hydrogels, by cryoSEM techniques, as well as ones which are a mixture of RADA16-I and an active sequence.

Rheological assays were performed to examine the viscoelastic properties of the newly synthesized hybrids. These results show that the additional motif hinders the formation of a β-sheet, and what follows, namely the self-assembling process, as previously reported by *Genové et al.*^37^. The authors observed that the longer additional peptide motif attached to the RADA16-I sequence is, the lower the viscoelasticity is. The results of CD studies are in agreement with this observation showing a decrease in the intensity of mole residue ellipticity at 216 nm (Fig. 3). The lower signal of intensity of β-sheet, which corresponds to their concentration, the less molecular interactions present in RADA16-I hybrids. The less molecular interactions, the less its gelation properties. Extending the length of sequence and thus disturbing of these interactions change its reological properties.

Many articles have reported that RADA16-I hydrogels have a fragile nature^24,37,71^. Scaffolds with appropriate rheological properties are selected depending on the application. Bone cements (like poly(methyl methacrylate) have G’ values round 10^4^–10^5^ Pa^89^ to strongly support bone cavities while, on the other hand, collagen matrices with a G’ value of ~ 4–60 Pa, exhibit excellent support for 3D tissue human fibroblasts^90^. The hybrids used here behave similarly to RADA16-I under shear stress, behaving like liquids over 4 Pa value. This property is necessary to keep cells in suspension in the gel. Application of a certain shear stress results in a decrease in these properties, presumably due to disruption of the hydrogen bonds present between amino acids in the RADA16-I chains. In order to assess a thixotropic nature of our hybrids a few more rheological experiments has to be done. PuraStat® is commercially available as a application in a syringe to suppress small haemorrhage. We have proved that after sonication the hydrids spontainuosely reassemble, which is normally considered as a proof of returning to its viscoelastic properties after exposure to shear stress. However we cannot be sure if they can reassemble after the pressure of syring and be appropriate for eg. 3D printing or applications similar to PuraStat®. They can be considered as extremely soft, so maybe naming them in this case, a colloid paste would be more suitable.

We established that the RADA peptides do not undergo self-degradation when they are diluted in water, which has been known to happen in some cases^91^. This is an important feature in terms of the production and shelf-life of commercially available wound dressings.

Experiments to determine stability in plasma were conducted with diluted gels (0.1 mg/ml). These experiments aimed to assess if the peptides can be easily degraded and cleared from the wound site. Albumins can be responsible for the absence of RADA hybrids in plasma, which was shown in our experiments. Albumins are known to be good transporters^92,93^, and all RADA hybrids have a strong affinity to them. It has previously been reported that RADA16-I scaffolds are biodegradable by various proteases^94^ and are thus biocompatible. The experiments proved that non-gelled hybrids are quickly eliminated. Due to their nature, they are probably hydrolyzed into amino acids. The quadruple Arg-Ala-Asp-Ala sequence is responsible for turning the peptide into a gel to stabilize the active sequence against enzymatic degradation and extend the release of the peptide by slowing down its degradation. Such strategies have been successfully applied previously^21^. The decay occurs at the junction of two phases, namely gel and plasma. The gel is penetrated by the plasma over time, and is digested at a much slower pace than the non-gelled peptide.

The proliferation of skin cells has a crucial impact on wound healing^95^. Two types of human cells were used in these experiments: HaCaT keratinocytes and 46BR.1N fibroblasts have proved to be a reliable model for preliminary analysis of potential wound healing stimulators^69^. Therefore, in this research, the proliferation of 46BR.1N fibroblasts and HaCaT keratinocytes was examined with an XTT test. In addition, when testing new biomaterials for tissue engineering applications, it is essential to determine their biocompatibility and check for any potential toxic effects on the human organism^20^. Analysis of cytotoxicity was thus performed with an LDH assay. The obtained results showed that the designed peptides supported the proliferation of human skin cells and did not show significant cytotoxicity towards these cells. This information indicates that they can be applied in vivo into a wound site without the risk of causing damage to skin cells and could be promising candidates for stimulation of wound healing. According to literature data^35,96^, RADA16 hydrogels can also promote cell migration. For example, *Bradshaw et al.* showed that RADA16-I with the collagen type I motif FPG enhanced migration of mouse embryonic NIH/3T3 fibroblasts, when compared to non-modified RADA16-I. Additionally, in their research RADA-FPG, when compared to RADA16-I, significantly decreased proliferation of fibroblasts and moderately decreased that of HaCaT keratinocytes. As cell migration is also very important for complete wound closure, it would be interesting to investigate the effect of the RADA16-I scaffolds used in these studies on migration of human cells, e.g. dermal fibroblasts or keratinocytes, in the future.

Considering the findings of *Kumada et al.*^35^ that RADA-16 hydrogels can support the growth of human ligament fibroblasts, we decided to evaluate the effect of the RADA derivatives tested in our work on the viability of human dermal fibroblasts. In *Kumada’s* work, two short biologically active peptide motifs, namely a 2-unit RGD-binding sequence PRG (PRGDSGYRGDS) and a laminin cell adhesion motif PDS (PDSGR) were coupled with RADA16-I. They showed that the designed scaffolds could enhance proliferation and migration of human periodontal ligament fibroblasts and the production of collagen type I and III in cell culture. This indicates that they might be used to reconstruct periodontal tissue. The experiment was demanding to perform due to the cells sinking into the gel and being difficult to observe under a microscope. The most challenging part was to visualize cells on the RADA-IM scaffold. Our results showed that after 3 days of incubation on the scaffold, human fibroblasts remained viable and showed a characteristic spindle-shaped morphology. These results indicate that the designed hydrogels support the growth of human fibroblasts and may serve as scaffolds for these cells in wound healing.

The utilization of RADA16-I based scaffolds in regenerative medicine, e.g. in nerve, brain injury, bone and wound healing, has been proved in the literature^59,69,71,97,98^. RADA16-I was tested as a wound dressing in a rat deep second-degree burn model. The results showed that RADA16-I could support wound healing because it reduced inflammation and oedema, accelerated wound closure, and increased more aesthetic and functional tissue^99^. Many different variations of RADA16-I hydrogel with different functional peptide modifications have been successfully tested in vitro and in vivo. Studies have shown that it provided axon regrowth across lesions studied in a sciatic nerve defect model in rats by appending it with a functional motif containing a cell adhesion the RGD tripeptide and neurite outgrowth peptide IKVAV^71^. It has been shown that a hydrogel modified with a sequence derived from bone morphogenetic protein-2 (BMP-2), namely KIPKASSVPTELSAISTLYLDDD, induces ectopic bone formation, which was certified by the experiments with implantations in the quadricep region of the thighs of Wistar rats^100^. It can also be conjugated with a peptide for promoting angiogenesis (KLTWQELYQLKYKGI), which shows the formation of new capillary vessels in chick embryos^101^. In addition, studies by *Wang et al.*^72^*.* proved that RADA-PRG (a mixture of RADA and PRG) could be utilized in tissue engineering to repair skin defects and serve as a scaffold for stem cell transplantation. This scaffold promoted the proliferation of skin-derived precursors (SKPs) and enhanced their survival and expression of hair induction genes as well as hair follicle neogenesis in vivo. RADA16-I peptide itself has been claimed to have a positive effect on angiogenesis^16,102^ and to promote regeneration in a variety of different tissues such as the nervous system^30,103^, mucosa^104–106^, periodontal defects^34^, bone^107^, and skin^99^. In our experiment, the improvement of skin wound healing was most remarkable following the application of the RADA-GHK and RADA-KGHK peptide hybrids. In animals treated with these hybrid peptides wound closure began earlier; it was observable from day two post-injury and was more significant than in groups treated with RADA16-I or RADA-IM (Fig. 14A,B). The initial enlargement of injuries in the first couple of days in RADA16-I mice wounds is commonly observed phenomenon in both dorsal skin injuries as well as ear pinna injuries. It is likely that this enlargement is a result of a macrophage-induced debridement of the wound area. However, on day eighteen post-injury, the wounds were closed in all groups. The difference between RADA-GHK/RADA-KGHK in comparison to RADA-IM can be explained simply by the differences between GHK/KGHK and IM bioactive fragments itself. GHK/KGHK have well established proregenerative properties while IM has been studied less extensively.

The results of histological examinations of tissues treated with many different variations of RADA16-I prove the complete reconstruction of the structure of healthy tissues. Earlier histological tests performed on damaged or burnt skin after administration of RADA16-I^99,108^ hydrogel showed complete restoration of the epidermis and dermis and, to a lesser extent, the hair follicles. However, following the application of RADA hybrid peptides, the restored tissue displayed remarkable characteristics manifested by formation of thick membrane-like structures covering the wounds; these were almost absent in mice treated with only RADA16-I (Fig. 15B). The differences in the membrane thickness might be a result of the increase in activity of fibroblast proliferation facilitated by the tested hydrogels. As shown in the proliferation tests on fibroblast cell lines all tested peptides improves proliferation of fibroblasts in wide range of concentrations. Although the concentration of peptides in hydrogel applied to the wound area is significantly higher than those examined in proliferation tests, the rigid tissue structure protects these cells from high concentrations of the peptide. Another characteristic feature of the wounds treated with the tested RADA peptides is the presence of multiple cells similar in morphology to muscle cells in the basal layer over the collagenous extracellular matrix. This observation suggests ongoing restoration of the muscle layer of the skin. Moreover, a comparison of our histological images of the skin with histological images of the skin treated with other hydrogels, such as fish scale collagen^109^ dextran hydrogel ^110^, hyaluronan‐gelatin^111^, chitin-PAA-GTMAC gel^112^ or SIKVAV-modified chitosan hydrogel^113^, also shows a similar image of reconstructed skin tissue. It is very interesting that the RADA-KGHK hydrogel, even as early as the 4th day after damage, had formed a thick film consisting of many layers of cells that separated the injury site from the external environment, thus protecting the wound against possible infection. Such a phenomenon has not been observed before for a RADA16-I hydrogel in skin tests^99,108^. It is also worth emphasizing that different hydrogels stimulate the growth of hair follicles to different degrees. It seems that the RADA-IM peptide hydrogel promotes their growth very effectively, which makes the reconstructed skin the most similar to healthy skin. However, it should be noted that further immunocytochemical tests are required in the future to investigate the high biological activity of the peptide hydrogels tested in this study.

## Conclusion

In summary, we have successfully constructed release systems based on a self-assembling RADA16-I peptide with three signal sequences that can be used to improve wound healing processes. We demonstrated that the hybrids form hydrogels, a β-structure, and fibres, can reassemble, can withstand shear stress, and their gels form a highly ordered structure. They are cleaved at the designated site, releasing the active motif, and can easily be cleared from plasma by hydrolysis. They are not cytotoxic, they stimulate skin cells to grow and they can serve as scaffolds for cells. In tests, the improvement of skin wound healing was most prominent following the application of the RADA-GHK and RADA-KGHK peptide hybrids, which is easily seen both from the wound closure kinetics and histology samples. Interestingly, RADA-IM, despite not stimulating wound closure as extensively as RADA-GHK and RADA-KGHK, stimulates the growth of hair follicles. These newly synthesized peptide hybrids showed great potential in wound healing. However, further research is needed.

## Supplementary Information

Supplementary Information.

## Data Availability

The raw/processed data required to reproduce these findings cannot be shared at this time due to technical or time limitations. These data are available from Sylwia Rodziewicz-Motowidło at the University of Gdańsk, Gdańsk Poland (s.rodziewicz-motowidlo@ug.edu.pl).
