# Supplementary material for: Release systems based on self-assembling RADA16-I hydrogels with a signal sequence which improves wound healing processes
Source: Sci Rep. 2023 Apr 18;13:6273. doi: 10.1038/s41598-023-33464-w (PMC10113214; doi:10.1038/s41598-023-33464-w)
Supplement: Supplementary file 1 — Supplementary Information. [file 41598_2023_33464_MOESM1_ESM.docx]

Release systems based on self-assembling RADA16-I hydrogels with a signal sequence which improves wound healing processes

Maria Dzierżyńska^1,#^, Justyna Sawicka^1,#^, Milena Deptuła^2^, Paweł Sosnowski^3^, Piotr Sass^3^, Barbara Peplińska^4^, Zuzanna Pietralik-Molińska^5^, Martyna Fularczyk^1^, Franciszek Kasprzykowski^1^, Jacek Zieliński^6^, Maciej Kozak^5^, Paweł Sachadyn^3^, Michał Pikuła^2^, Sylwia Rodziewicz-Motowidło^1,*^

^1^ Department of Biomedical Chemistry, Faculty of Chemistry, University of Gdańsk, Poland

^2^ Laboratory of Tissue Engineering and Regenerative Medicine, Department of Embryology, Medical University of Gdańsk, Gdańsk, Poland

^3^ Laboratory for Regenerative Biotechnology, Faculty of Chemistry, Gdańsk University of Technology, Gdańsk, Poland

^4^ NanoBioMedical Centre, Adam Mickiewicz University, Poznań, Poland.

^5^ Department of Macromolecular Physics, Faculty of Physics, Adam Mickiewicz University, Poznań, Poland

^6^ Department of Surgical Oncology, Medical University of Gdańsk, Poland

#These authors contributed equally to this work.

***** Correspondence: s.rodziewicz-motowidlo@ug.edu.pl (S.R-M.)

Supplementary data

*Table 1S. Peptides theoretical and experimental masses and retention times*

| Peptide ID | Theoretical monoisotopic mass [Da] | Experimental monoisotopic mass [Da] | Retention time [min]* |
| --- | --- | --- | --- |
| RADA-16-I | 1711.846 | 1711.858 | 2.769 |
| RADA-IM | 3209.621 | 3209.562 | 3.371 |
| RADA-GHK | 2657.324 | 2657.260 | 2.971 |
| RADA-KGHK | 2842.441 | 2844.370 | 2.909 |

*UHPLC conditions 5-100% B in 15 min, where A – 0.1% TFA in water, B – 80% acetonitrile in water with 0.1% TFA, Kinetex 2.6 µm C8 100Å, 2.1x100 mm

*
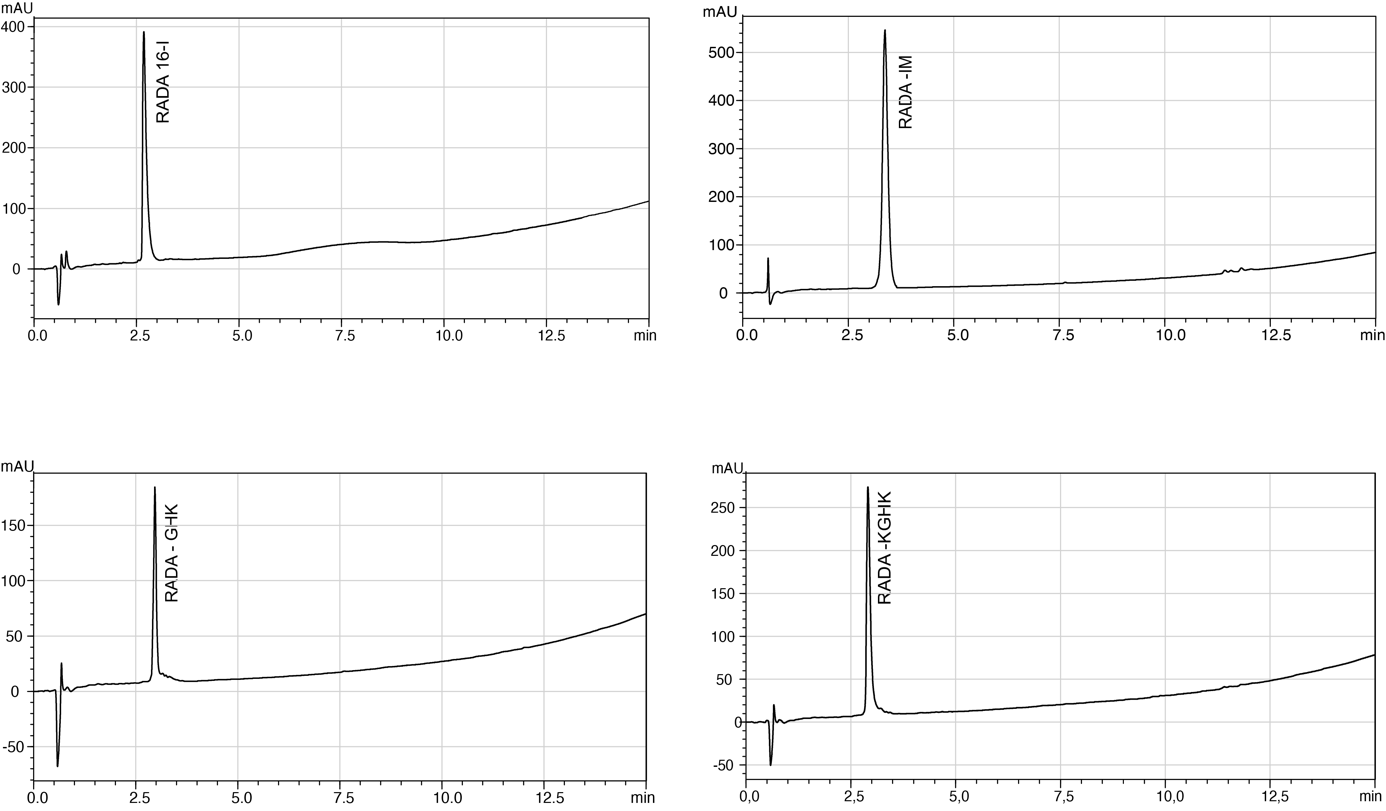
*

*Figure 1S. Chromatograms of pure RADA hybrids.*

*Table 2S. Masses of RADA hybrids observed during ESI ionization*

|  | +3 | +4 | +5 | +6 |
| --- | --- | --- | --- | --- |
| RADA-16-I | 428.9563 | 571.6243 |  |  |
| RADA-IM |  | 803.6498 | 643.1198 | 536.0914 |
| RADA-GHK |  | 665.6063 | 532.6763 | 444.0661 |
| RADA-KGHK | 945.8053 | 711.8451 | 569.6920 |  |

*
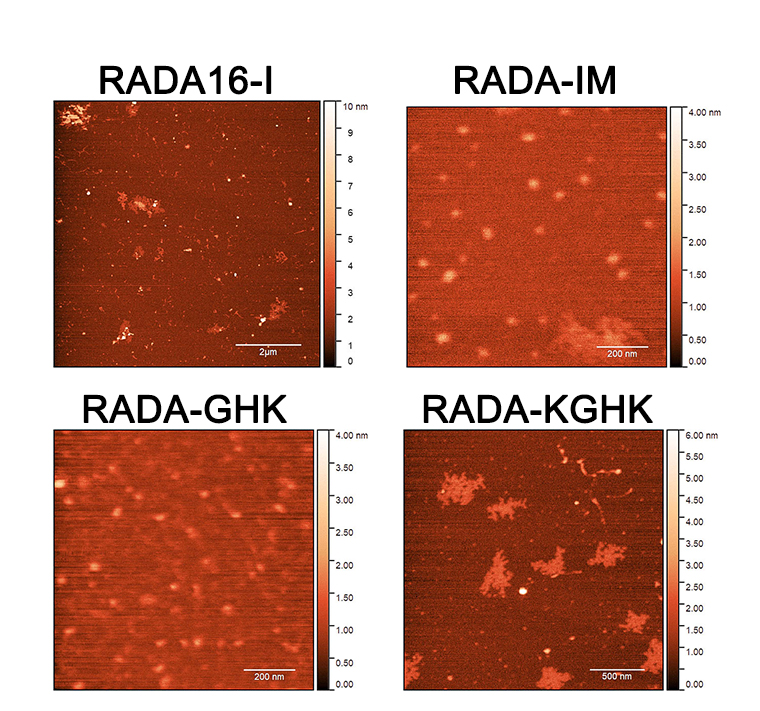
*

*Figure 2S. AFM images of RADA16-I and RADA hybrids after sonication.*

*
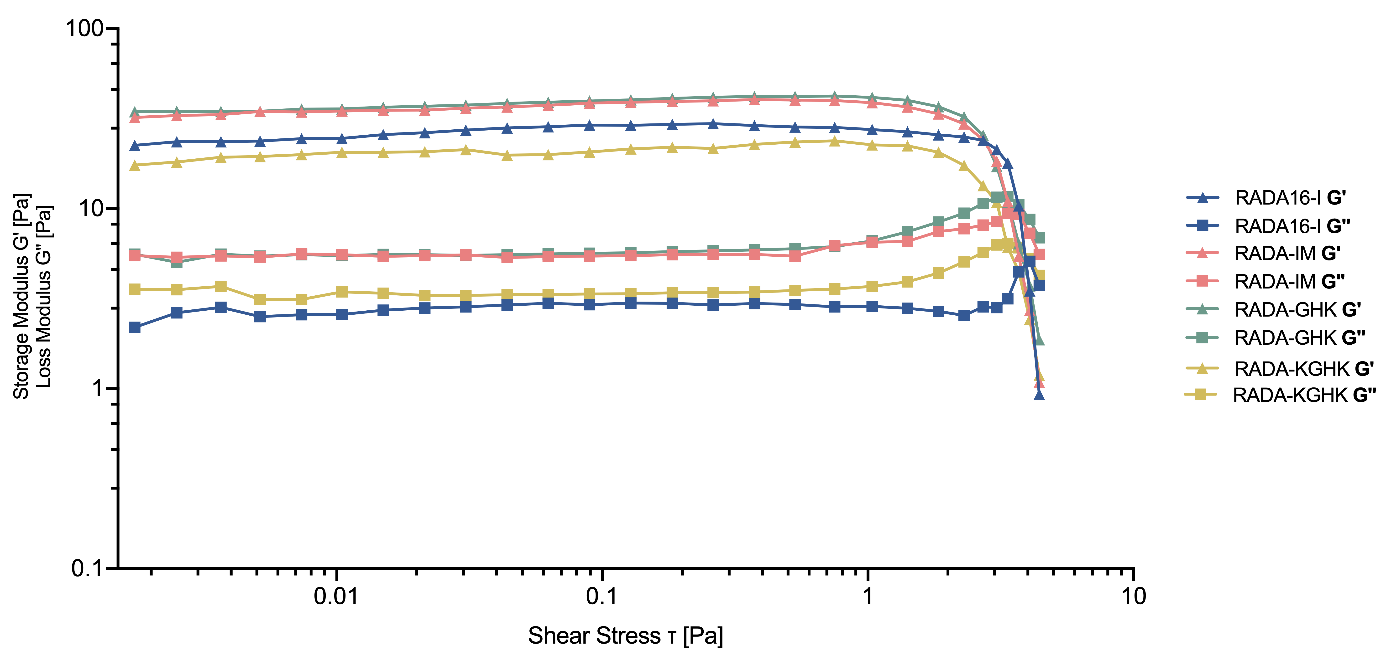
*

*Figure 3S. G’ and G” of RADA16-I hybrids as a function of shear stress at ω=6.28 rad/s and 37°C.*


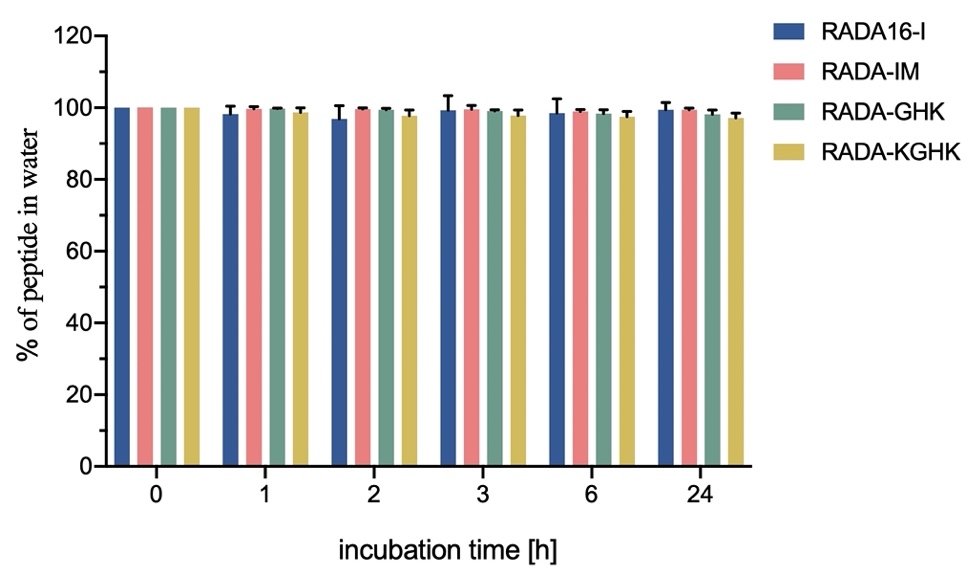


*Figure 4S. Stability of peptides in aqueous solution. The graph shows results from 3 independent experiments. Results are presented as mean with SD.*

*Table 3S. Most abundant signals in mass spectrometry corresponding to identified fragments of peptides after elastase digestion.*

| PEPTIDE | Sequence | Mass [Da] |
| --- | --- | --- |
| RADA-IM | Ac-(RADA)_4_-GGG-AAPV-COOH | 2222.08 |
|  | NH_2_-GGRDKVYR-NH_2_ | 948.49 |
|  | NH_2_-GGGRDKVYR -NH_2_ | 1005.51 |
| RADA-GHK | Ac-(RADA)_4_-GGG-AAPV-COOH | 2222.03 |
|  | NH_2_ -GHK-NH_2_ | 339.93 |
| RADA-KGHK | Ac-(RADA)_4_-GGG-AAPV-COOH | 2222.03 |
|  | NH_2_-PV-GGG-KGHK-NH_2_ | 834.45 |
|  | NH_2_-APV-GGG-KGHK-NH_2_ | 905.47 |


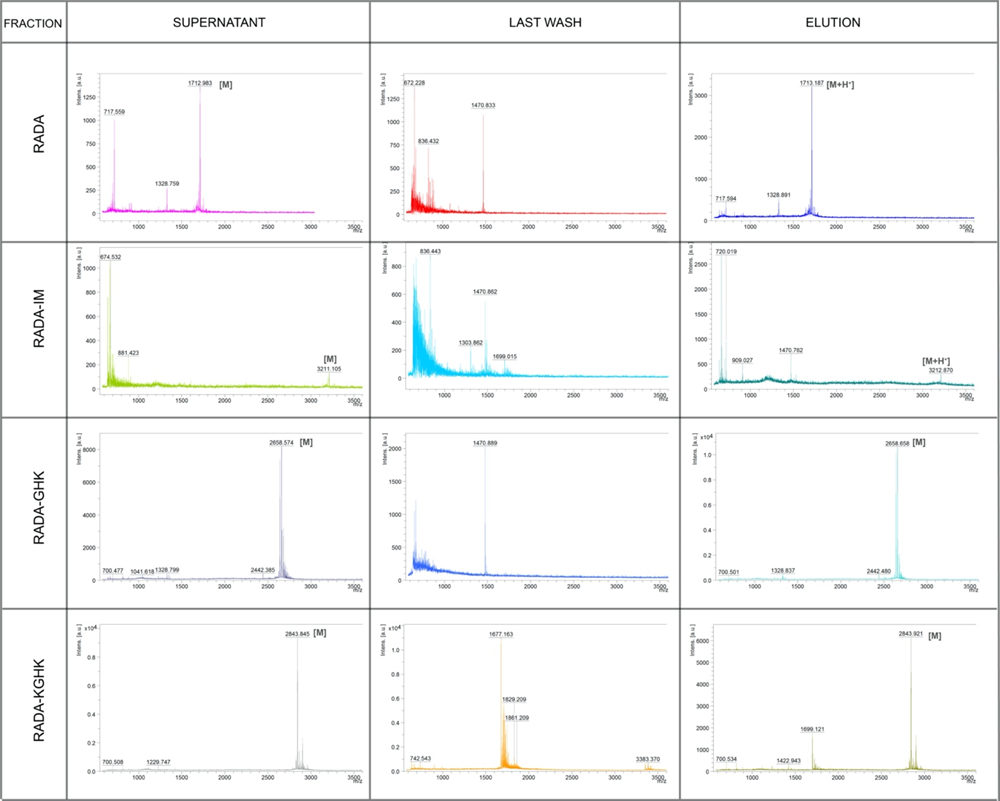


*Figure 5S. Affinity studies of RADA hybrids.*

*
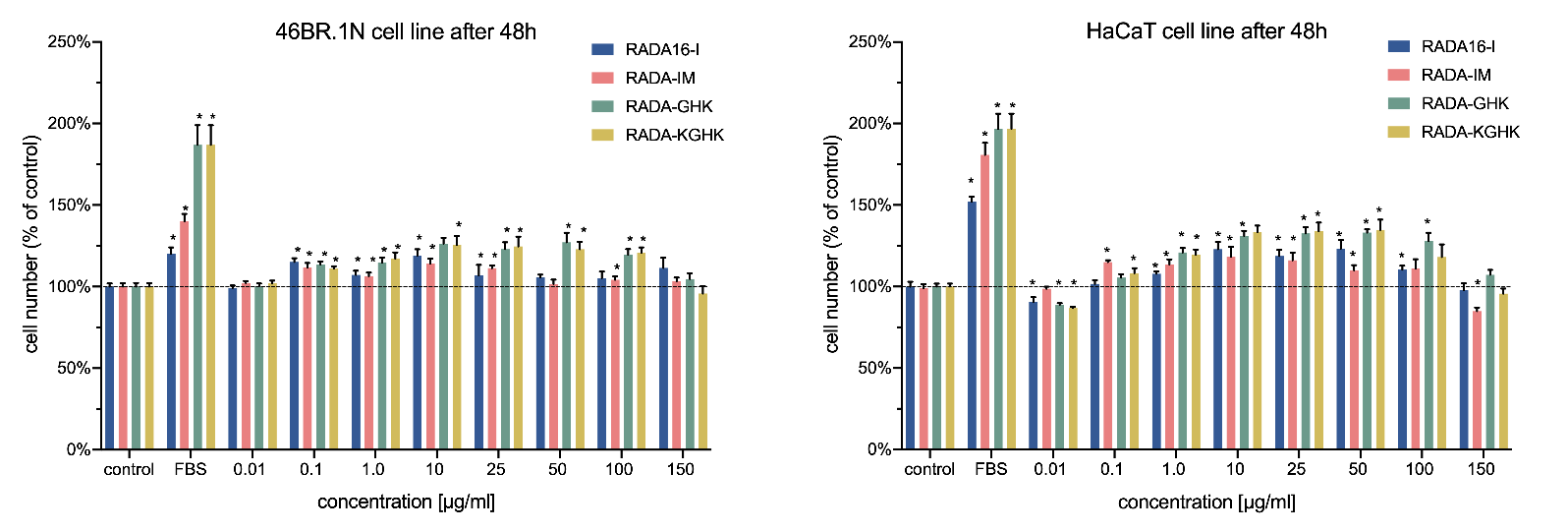
*

*Figure 6S. RADA hybrids proliferation test for 46BR.1N fibroblasts and HaCaT keratinocytes after 48h. The graph shows results from 3 independent experiments (4 replicates in each, n=12). Results are presented as mean with SD. *- statistically significant differences compared to control, Mann-Whitney U test, p<0.05. FBS - positive control - cells grown in medium containing 10% FBS.*

*
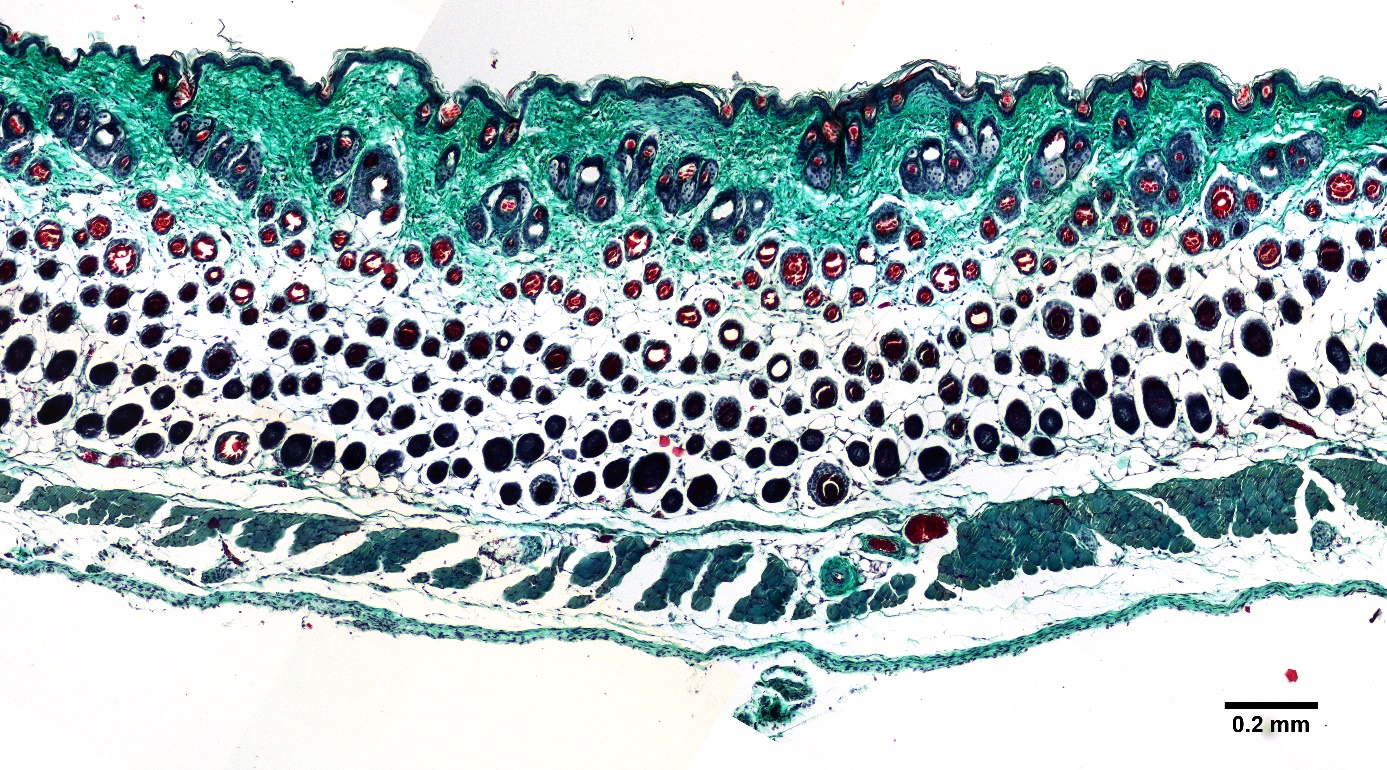
*

*Figure 7S. Histological image of healthy skin.*
